# Machine Learning-Driven Optimization of Continuous-Flow Photoredox Amine Synthesis

**DOI:** 10.1021/acs.oprd.4c00533

**Published:** 2025-05-21

**Authors:** Perman Jorayev, Sebastian Soritz, Simon Sung, Mohammed I. Jeraal, Danilo Russo, Alexandre Barthelme, Frédéric C. Toussaint, Matthew J. Gaunt, Alexei A. Lapkin

**Affiliations:** † Department of Chemical Engineering and Biotechnology, 2152 University of Cambridge, Cambridge CB3 0AS, United Kingdom; ‡ Astex Pharmaceuticals, 436 Science Park, Cambridge CB4 0QA, United Kingdom; § Cambridge Centre for Advanced Research and Education in Singapore, 563725 CARES Ltd., 1 CREATE Way, CREATE Tower #05-05, Singapore 138602, Singapore; ∥ Department of Chemical Engineering, Materials, and Industrial Production, University of Naples Federico II, Piazzale V. Tecchio 80, Naples 80125, Italy; ⊥ 219947 UCB Pharma S.A. Allée de la Recherche, Brussels 60 1070, Belgium; # Yusuf Hamied Department of Chemistry, 2152 University of Cambridge, Cambridge CB2 1EW, United Kingdom

**Keywords:** Bayesian optimization, photoredox chemistry, flow chemistry, automation

## Abstract

Photoredox catalysis plays an important role in the synthesis of pharmaceutically relevant compounds such as C­(sp^3^)-rich tertiary amines. The difficulty of identifying underlying mechanistic models for such novel transformations, coupled with the large reaction space of this reaction class, means that developing a robust process is challenging. In this work, we demonstrate the machine learning-driven optimization of a photoredox tertiary amine synthesis with six continuous variables (e.g., concentration, temperature, residence time) and solvent choice as a discrete variable, in a semiautomated continuous flow setup. Starting with a large library of solvents, the workflow included multiple steps of *a priori* knowledge generation (e.g., solubility predictions) to narrow the discrete space. A novel Bayesian optimization algorithm, nomadic exploratory multiobjective optimization (NEMO), was then deployed to identify and populate the Pareto front for the two reaction objectives—yield and reaction cost. Permutation feature importance and partial dependence plots identified the most important parameters for high yield, sig3, the asymmetry of the s-profile for the discrete space, and equivalences of alkene and Hantzsch ester for the continuous variables. Catalyst loading and residence time were found to be correlated to absorbed photon equivalence, while catalyst loading was additionally the main parameter to drive cost. Even though productivity was not an optimization objective, the best result achieved in flow was ∼25 times higher than reactions in batch, which equals to ∼12 g per day throughput.

## Graphical abstract

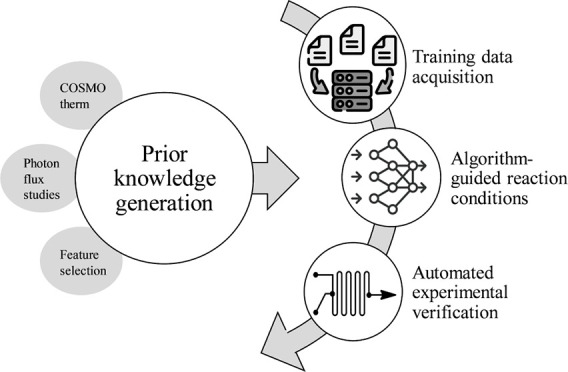

## 1 Introduction

Recently, visible-light photocatalysis has seen increased adoption in academia and industry, especially in the field of continuous flow chemistry in plug or laminar flow reactors, owing to its ability to streamline access to pharmaceutically relevant compounds under milder conditions, which would normally require lengthy multistep syntheses.^1^ However, while the use of flow reactors allows for faster reaction times and more efficient process control compared to their batch alternatives, holistic and robust process development of such processes for novel chemical transformations is still a laborious and complex task. This is mostly due to the difficulties in identifying the underlying chemical and physical parameters that affect the process objective(s), quantifying the nonlinear interactions between them, and the lack of prior experimental and computational data. These difficulties necessitate the development of a workflow to (i) efficiently generate *a priori* knowledge (such as optimal reactor and lamp choices, solubility predictions, and featurization of discrete variables with relevant molecular descriptors) and (ii) use said knowledge to efficiently find the trade-off curve of competing process objectives (i.e., the Pareto front).

The advancements in the field of photocatalysis have mainly been triggered by a deeper understanding of the underlying mechanisms, a wider range of photocatalysts, and improved light-emitting diode (LED) technology in terms of energy efficiency, cost, and temperature control. Since the first reported application of photoredox catalysis in organic chemistry more than 40 years ago,^2^ the field has significantly expanded with the recent years.^3,4^ The main focus was on the discovery of novel molecules and transformations, which were often performed in batch, leaving a significant gap between the discovery of new transformations and the development of robust and scalable processes. Such batch experiments, though convenient and easier to set up, usually require long reaction times (10–50 h), may not be easily reproducible between research laboratories due to differences in physical setups, and are difficult to automate and scale when reactions are multiphase and include complex physical interactions, such as light attenuation in different media.

Highlighted by Noël and coworkers as good reasons for continuous-flow photocatalysis, transferring results from batch to continuous-flow systems allows for reduced reaction time (due to improved irradiation of the reaction solution), easier scalability, precise control of time and temperature, reproducibility, and faster mixing, among others.^5^ This ability to control and accurately tune the physical parameters helps avoid side reactions and makes it easier to conduct multistep and multiphase reactions.^6^ It was also shown that if physical processes of light attenuation and quenching of active species are accounted for, a relatively straightforward optimization allows to find the range of optimal designs for photochemical flow reactors.^7^ A number of successful lab-scale demonstrations of continuous photochemical syntheses were published. For example, Seeberger and colleagues demonstrated the dehalogenation of α-chlorophenacylacetates in a continuous-flow microreactor using Ru­(bpy)_3_Cl_2_ as a photocatalyst.^8^ Compared to the results in batch (<50% yield in 24 h), the authors achieved an 82% yield under 30 min in flow. Similarly, Noël and coworkers developed a continuous-flow microreactor for trifluoromethylation and perfluoroalkylation of five-membered heterocycles. Using gaseous CF_3_I for the trifluoromethylation reaction, all substrates were converted to their respective products in good yields (55–95%) in 8–16 min (vs. 12–72 h in batch).^9^

To transfer any new photochemical transformation from the discovery stage batch protocols to continuous flow, it is desirable to assemble knowledge about the system in the form of models. Access to a good model may allow for the *in silico* design of flow reactions.^10^ However, as the development of first-principle mechanistic models of organic synthetic reactions is rather challenging and slow, recent focus in reaction development/optimization has increasingly on using “self-optimizing” experimental systems (automated experimental systems able to perform optimization of a reaction and, potentially, also separation, without human intervention) that are able to generate the required data and perform reaction optimizations with data-driven statistical methods, as opposed to mechanistic models.^11^ When optimizing for a single objective over continuous variables only, several optimization algorithms such as Nelder–Mead Simplex,^12^ SNOBFIT,^13^ and steepest descent^14^ have been implemented for different types of reactions, such as nanoparticle synthesis^15^ and heterogeneous catalytic reactions.^16^ Without a prior model of the chemical reactions, these “black box” approaches focus on relationships between input and output variables or objective(s). These algorithms have some significant limitations: they are either local search algorithms with limited scalability to larger systems (e.g., Nelder–Mead Simplex),^17^ require expensive derivative estimations and are inaccurate for noisy systems (e.g., steepest descent), or have slow convergence despite successful implementations as a global search algorithm (SNOBFIT).^18,19^ Moreover, most of the published works on self-optimization are limited to optimizing up to five continuous variables for a single objective.^20^

The more advanced versions of self-optimization methods are based on Bayesian optimization (BO) algorithms, such as MOAL,^21^ TS-EMO,^22^ MVMOO,^23^ and Google Vizier,^24^ which construct nonparametric statistical models, so-called Gaussian processes, using a sequential active learning approach (i.e., retraining a model when new experimental observations become available). BO approaches utilize all available data to build statistical models and are thus data-efficient and can be used to optimize for multiple, sometimes competing, process objectives (multiobjective optimization). The models provide quantification of uncertainty that is used to solve the inherent exploration–exploitation trade-off within derivative-free optimization. Extensions of TS-EMO algorithms have been successfully implemented for multiobjective self-optimization^25–27^ and solvent selection.^28,29^ Single-objective BO has been effectively demonstrated for optimizing the scope of organic reactions, including both continuous and discrete variables, e.g., in ref 30. Examples of the use of multiobjective optimization in so-called “self-driving labs” can be found in the works of Torres et al., with the optimization of a nickel- and photoredox-catalyzed cross-electrophile coupling,^31^ Sagmeister et al., with the optimization of the 2-step synthesis of edaravone;^32^ or Zhang et al., with an in-depth look into the optimization of a Schotten–Baumann reaction.^33^ These works feature traditional, multiobjective closed-loop optimization, without the formalized use of *a priori* knowledge in its optimization processes, which will be discussed in more detail below. Additionally, a more complete overview on self-driving laboratories can be found in the excellent review by Aspuru-Guzik and coworkers.^34^

When expanded into discrete variables, including solvents, reagents, or ligands, most of the self-optimization algorithms mentioned above struggle with the “curse of dimensionality” (a poor scaling of performance of computational methods with the number of parameters) and inefficiencies (in predictive accuracy) of black-box optimization algorithms. A common approach to overcome this problem has been demonstrated by using molecular descriptors to map the discrete variables onto a continuous space. Parameterization of discrete variables with relevant descriptors could increase the predictive accuracy of surrogate models and unlock new mechanistic insights. Earlier work in describing the partition behavior of solutes in solvents was demonstrated by Zissimos et al., comparing experimental Abraham descriptors with Klampt’s COSMOments, often used for quantitative structure–property relationship (QSPR) and linear free energy relationship (LFER), to explain solvation phenomena for a data set of 470 compounds.^35^ The authors found a large overlap of chemical information content between the two sets and that computationally calculated Klampt descriptors could be used to replace the experimentally calculated Abraham parameters. In comparison, Ševčík et al. used 17 descriptors as input and were only able to predict the experimental Abraham parameter *S* using multiple linear regression modeling.^36^

The use of Abraham parameters for solvent selection in reactions, instead of QSPR, was demonstrated by Adjiman and colleagues in a computer-aided molecular design (CAMD) study. Starting with six solvents in the training data set and a linear regression model, the authors found nitromethane to be the best solvent for maximizing the reaction rate constant in different solvents for the Menshutkin reaction after five iterations.^37^ Amar et al. expanded the descriptor space to 17 to optimize for high conversion (>90%) and diastereoselectivity (>60%) in an asymmetric hydrogenation reaction to synthesize brivaracetam, a new antiepileptic drug produced by UCB.^28^ While this approach worked well for identifying ideal solvent candidates, all of the continuous variables, such as temperature and concentrations of substrates, were kept constant during the solvent screening. Similarly, Zhang et al. used 15 descriptors for solvents in a Mitsunobu transesterification reaction to produce isopropyl benzoate.^29^ Using an autoencoder for dimensionality reduction and artificial neural networks (ANNs) as a surrogate model to design the experiments, the final surrogate model identified 1-chloropentane (93% yield) as a promising solvent for the single objective of maximizing reaction yield.

Even though the use of a larger number of descriptors could provide more information about solvents (for better differentiation), the concept of a “plethora of descriptors”^35^ comes at the cost of sampling from a high dimensional space and potentially introducing (reaction-) irrelevant descriptors. Dimensionality reduction techniques, such as principal component analysis (PCA) or an autoencoder, could reduce the descriptor space but with the risk of losing solvability and reaction-relevant descriptors.

We postulate the generic problem addressed in this work as “what is an effective methodology to develop robust synthesis procedures based on novel synthetic methods?”. Our hypothesis is that, to be effective, the methodology we are seeking should make use of a combination of (i) Bayesian active learning optimization, which has been proven to be effective with small experimental budgets, and (ii) an effective generation and assembly of *a priori* knowledge about the chemical system of interest, which will further constrain the experimental search space.

Here, we use *a priori* in a somewhat loose sense, meaning data about a reaction system that, in principle, could already be available for the specific molecules and the process system and needs to be assembled according to relevance. This relates to the concept of *process context* introduced by Lapkin and coworkers a decade earlier.^38^ Understanding context relevant to a specific reaction is equated to understanding the relevant chemical and physical phenomena and their interactions. This is critical in developing the best method of parameterizing the specific chemical reaction for machine learning-based reaction development workflows. In the absence of such knowledge, our models are trained on data that would likely not contain the relevant underlying phenomena, and hence the models would not reach the desired accuracy and predictive ability.

Of the many novel transformations developed over the past decade, we were attracted to a process involving a photocatalyzed multicomponent reaction to generate C­(sp3)-rich tertiary alkylamines from readily available starting materials,^39^ and we selected to demonstrate our methodology on this case study. A proposed mechanism for this transformation is shown in Scheme 1. The developed workflow is schematically shown in Figure 1. In the workflow, a machine learning-driven optimization of the amine synthesis was demonstrated with six continuous variables and 20 solvents, using a semiautomated continuous flow setup. Starting with a library of 115 solvents, the workflow includes multiple steps of *a priori* knowledge generation (e.g., solubility predictions and measurements and UV–vis and actinometry studies) to narrow the space of discrete choices in this reaction system. Once prior knowledge was assembled, we then used the NEMO algorithm to identify and populate the Pareto front for the two reaction objectives—yield and cost—in a semiautomated continuous flow optimization campaign. While demonstrated on a specific example of a challenging-to-scale synthetic chemical procedure, the approach of iterative identification of the relevant reaction context that aids and constrains active learning reaction optimization is generic. It is our further goal to develop an automated methodology for prior knowledge generation, which will be described elsewhere.

**Scheme 1:**
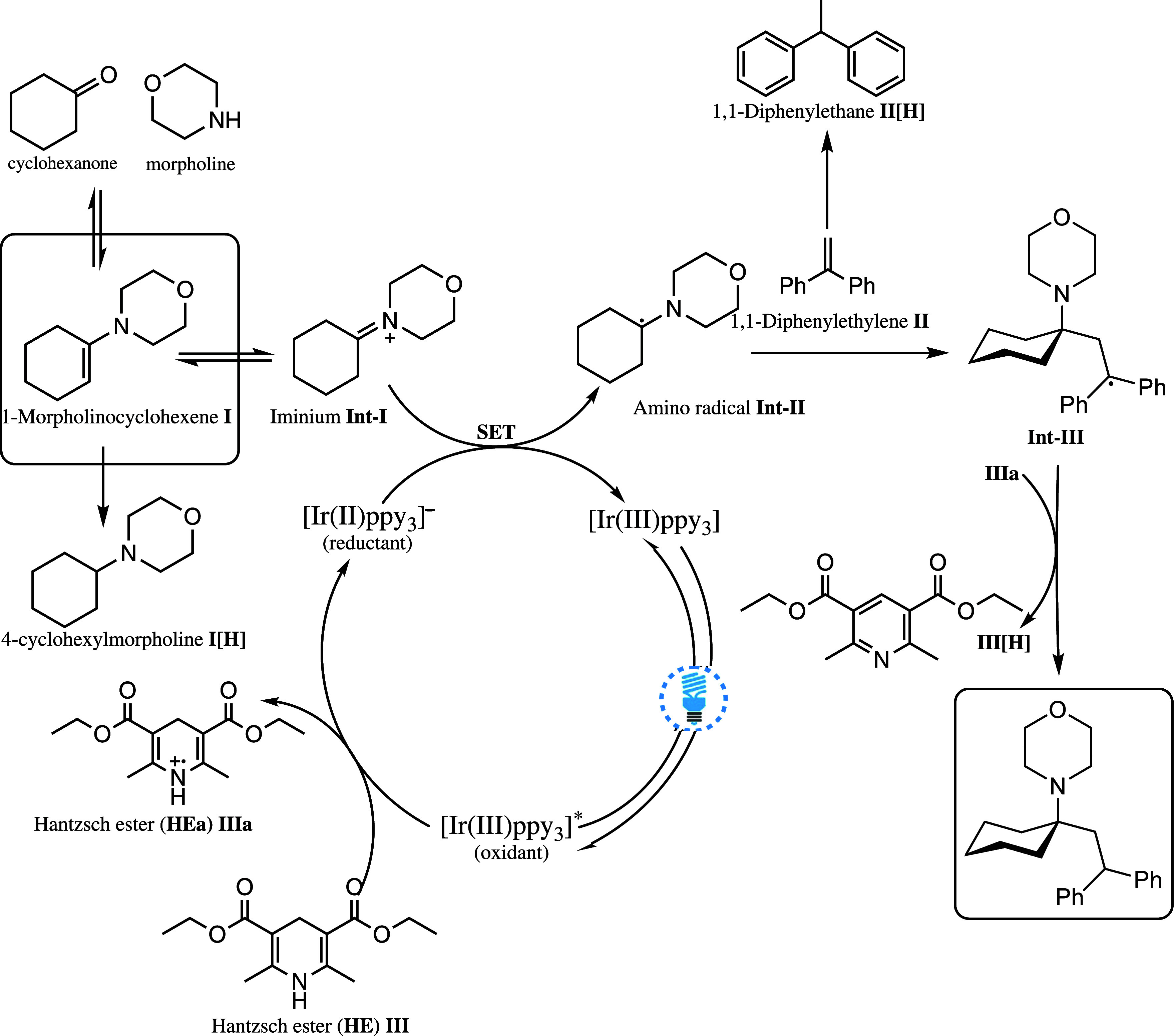
Proposed Mechanism for Ir-Catalyzed Photoredox Amine Synthesis in This Work, Adopted from Ref 39 ^a^

a Starting with 1-morpholinocyclohexene (**I**), the first step, possibly during the reaction mixture preparation, is acid-catalyzed iminium ion intermediate (**Int-I**) formation. Then, once visible light reaches the catalyst species, photocatalyst Ir­(ppy)_3_ is excited, leading to the long-lived photoexcited [Ir­(III)­ppy_3_]* oxidant (1.9 μs lifetime),^40^ which is quenched by Hantzsch ester (**III**) resulting in [Ir­(II)­ppy_3_]^−^, a reductant, and the associated Hantzsch ester radical cation **IIIa**. Combined with the iminium ion intermediate (**Int-I**), the species [Ir­(II)­ppy_3_]^−^ is sufficiently reducing to undergo single-electron transfer (SET) to regenerate the ground-state photocatalyst and amino radical intermediate (**Int-II**). Added to 1,1-diphenylethylene (**II**), **Int-II** leads to C–C bond formation to form a benzylic radical **Int-III**. Followed by hydrogen-atom transfer (HAT) with the Hantzsch ester radical cation **IIIa**, **Int-III** leads to the formation of the final tertiary amine product 4-(1-(2,2-diphenylethyl)­cyclohexyl)­morpholine.^39^

**Figure 1:**
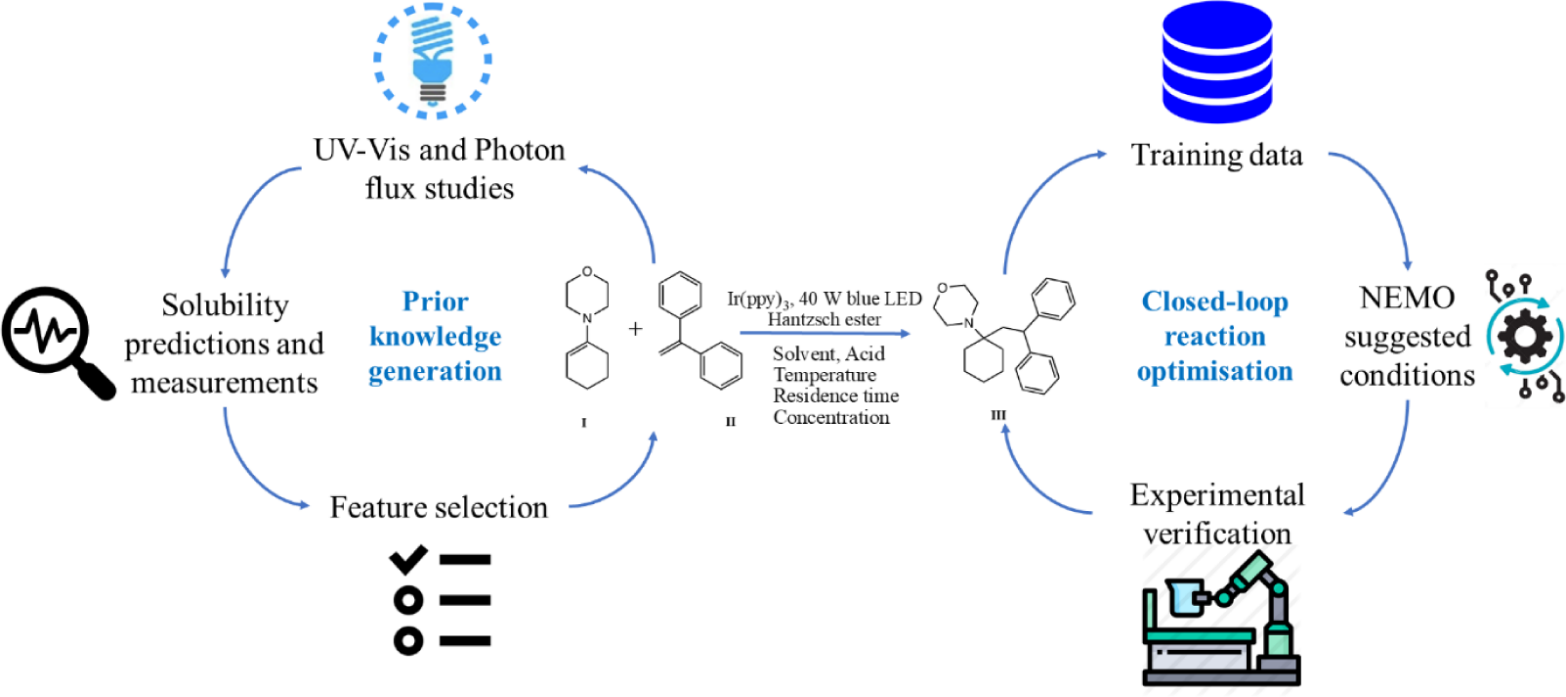
An overview of the workflow proposed for generating *a priori* knowledge and optimizing a reaction experimentally to develop a robust synthetic protocol.

The early version of this manuscript was published as a preprint.^41^

## 2 Methods and Materials

### 2.1 Materials

Reagents 1-morpholino-1-cyclohexene, >97%; 1,1-diphenylethylene, 98%; diethyl 1,4-dihydro-2,6-dimethyl-3,5-pyridinedicarboxylate, >98%; and tris­(2-phenylpyridinato)­iridium­(III), >98% were purchased from TCI Chemicals and used as received. 1,2-Dichloroethane, anhydrous, 99.8%; 1,3-dimethyl-2-imidazolidinone, absolute, ≥99.5%; 1,3-dimethyl-3,4,5,6-tetrahydro-2­(1*H*)-pyrimidinone, absolute, ≥99.0%, furfuryl alcohol, 98%; tetrahydrofurfuryl alcohol, 99%; 2-methyltetrahydrofuran, anhydrous, ≥99.0%; 2-propanol, anhydrous, 99.5%; acetonitrile, anhydrous, 99.8%; benzyl alcohol, anhydrous, 99.8%; *N*,*N*-dimethylformamide, anhydrous, 99.8%; dimethyl sulfoxide, anhydrous, ≥99.9%; cyclohexanone, *ReagentPlus*, 99.8%; ethanol, anhydrous, ≥99.5%; ethyl acetate, anhydrous, 99.8%; *N*,*N*-dimethylacetamide, anhydrous, 99.8%; 1-methyl-2-pyrrolidinone, anhydrous, 99.5%; acetone, HPLC plus, ≥99.9%; tetrahydrofuran, anhydrous, ≥99.9%; dichloromethane, anhydrous, ≥99.8%; propionic acid, ACS reagent, ≥99.5%; naphthalene, 99%; mesitylene, 98% were purchased from Sigma–Aldrich and used as received. Nonanhydrous solvents were bubbled with nitrogen for 45 min and stored over activated molecular sieves in a Schlenk tube. For a fair comparison, the cost of solvents was calculated based on the 2 L solvent cost (unless not available from a supplier) in the cost objective function.

### 2.2 Procedures

Experiments were carried out in flow using a Vapourtec R2 Series with a 10 mL UV-150 reactor. Given the solubility and mixing issues highlighted in Section 3, individual reaction solutions were prepared inside a glovebox before each reaction and loaded directly from the vial using a Gilson 271 Liquid Handler. The solids—Hantzsch ester (HE, diethyl 1,4-dihydro-2,6-dimethyl-3,5-pyridinedicarboxylate), catalyst (*fac*-Ir­(ppy)_3_), and internal standard (naphthalene, 0.02 mmol)—were stored in the glovebox, and the relative amounts were added to a 10 mL (crimp) vial. 0.2 mmol (34 mL) of 1-morpholino-1-cyclohexene **I** was added with relative amounts of 1,1-diphenylethylene **II** (0.2–0.4 mmol), propionic acid (0.02–0.2 mmol), and the respective anhydrous solvent to prepare a 5 mL final solution. The vial was closed, wrapped with parafilm, and transferred to a sonicator bath to promote faster dissolution of the solids (<5 min). Before pumping the solution through the reactor, an inert nitrogen line was connected to the vial to replace the pumped solution and avoid air intake. Reactions were conducted in a UV-150 reactor with a 470 nm LED lamp and a 6 bar BPR for the given residence time and temperature. An external chiller was used to achieve lower temperatures. The crude reaction was collected, and 25 mL of this mixture was diluted to 1.0 mL in acetonitrile and analyzed using HPLC. The experimental setup is given in Figure S1 and full data generated in the optimization is given in Table S6. The reproducibility of the setup for reaction yield estimation was calculated to be around 1.8% (Table S7).

### 2.3 Analytical Methods

The amount of HE individually dissolved in 38 different solvents was measured by using a Magritek Spinsolve 60 ULTRA Benchtop NMR with mesitylene as an internal standard. The analysis was conducted using 1D EXTENDED+ at 2 scans, a 6.4 s acquisition time, a 2 min repetition time, and a 90° pulse angle.

The reaction composition was analyzed using HPLC (Shimadzu LC-20A, D2 lamp with PDA detector; Eclipse Plus C18, 95 Å, 3.0 × 100 mm, 3.5 mm column). An injection volume of 5 mL, oven temperature of 30 °C, and total flow rate of 1.0 mL min^–1^ with acetonitrile/water (45%/55%) were found to be optimal. The acetonitrile concentration was increased to 80% in 17 min, then to 98% in 1 min, held at 98% for 1 min before reducing it to 50% in 1 min and holding it at 50% for 1 min. The product vs. internal standard calibration plot is provided in Figure S2.

### 2.4 The Nomadic Exploratory Multiobjective Optimisation Algorithm

We have recently developed a nomadic exploratory multiobjective optimization (NEMO) BO algorithm to efficiently optimize complex black-box systems for multiple objectives over continuous and discrete variables simultaneously (see Figure 2).^42^ Unlike other BO algorithms that employ a single surrogate model (often Gaussian processes), NEMO fits several black-box models—Gaussian processes, neural networks (concrete, Bayesian, etc.), XGBoost, and NGBoost—on the training data for each black-box objective. When beginning an optimization campaign, Latin hypercube sampling (LHS),^43,44^ a statistical method used to sample from a given space subject to the condition of sampling a single point in a given row and column, is used to generate the (next) experimental conditions to carry out. Once the surrogate models are trained on the training data set, the model with the best prediction accuracy from step 1 (e.g., XGBoost) is used to predict the outcomes of LHS-sampled points. In our implementation, five conditions were selected per iteration to be verified experimentally to accelerate the optimization process. Compared to some other BO algorithms, which would select the top five conditions based on the highest expected hypervolume improvement (EHVI) of each individual point, which could result in all five points being close to each other, NEMO optimizes for the highest EHVI for the combination of points. Once a combination of multiple conditions is selected, NEMO utilizes the SciPy minimize function, which uses the L-BFGS-B algorithm, to refine the identified points to further increase EHVI. The sampled conditions with the predicted outcome and the associated uncertainty account for the exploration and exploitation trade-off for development of a robust and predictive model, instead of potentially getting stuck at and exploiting a local optimum. Finally, the suggested conditions are verified experimentally and fed back to the algorithm until the defined optimization criteria are achieved. It is important to highlight that training, hyperparameter optimization, model selection, and sampling of the best conditions are fully automated within NEMO. NEMO has recently been included in the package AMLearn, which also includes hardware integration for the rapid deployment of self-optimization systems.^45^

**Figure 2:**
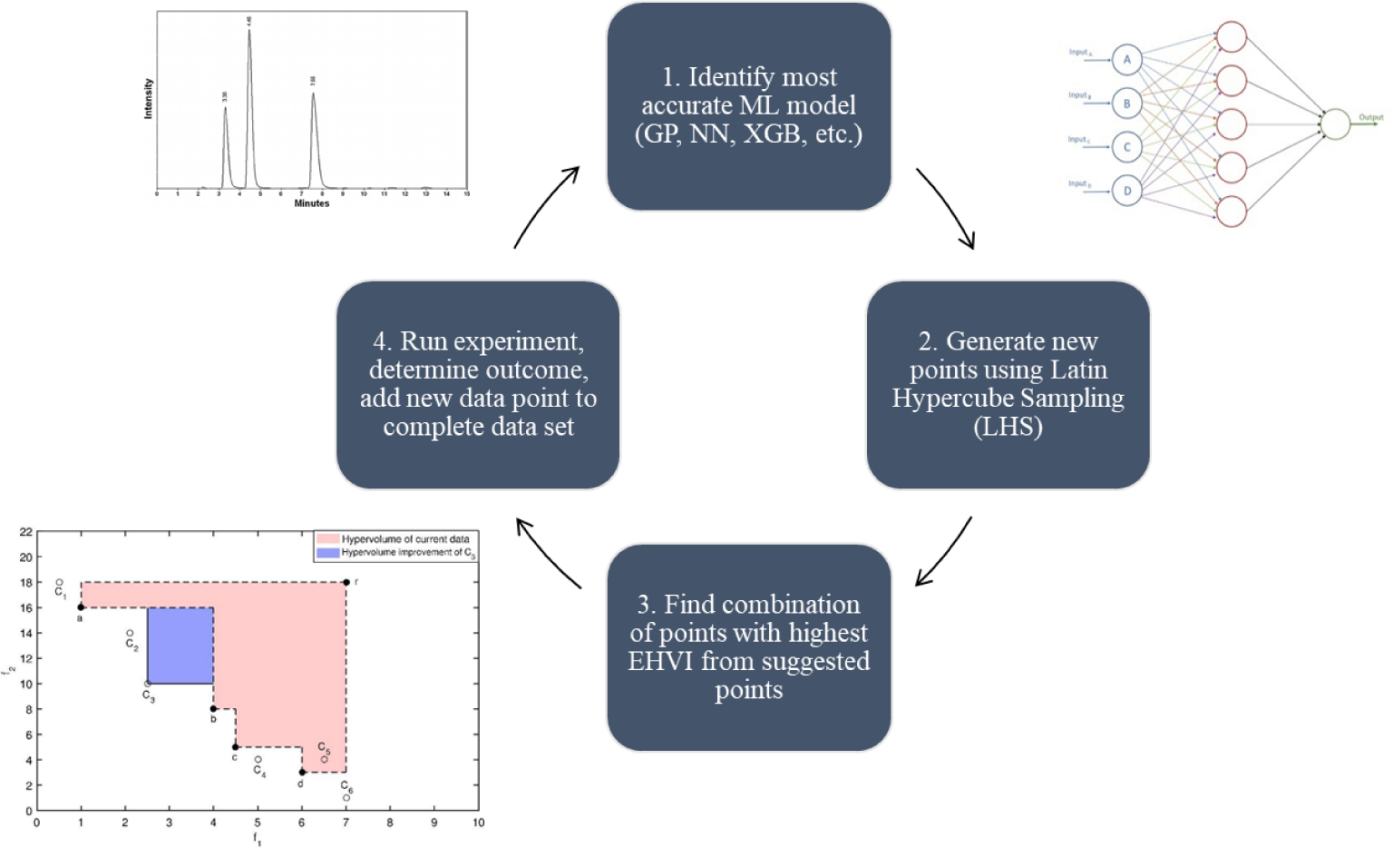
An overview of the NEMO algorithm.

## 3 Results and Discussion

### 3.1 Prior Knowledge Generation

#### 3.1.1 Identification and Parametrization of the Reaction Space

The lower and upper bounds for the six continuous reaction variables are shown in Table 1. 1-Morpholine-1-cyclohexene **I** (40 mM) was used as the limiting reactant alongside 1.0–2.0 equiv of 1,1-diphenylethylene **II** and HE, based on the proposed reaction mechanism (Scheme 1).^39^ If the HE solubility in a particular solvent was less than 80 mmol/L (i.e., 2.0 equiv for HE), then that solubility value was used as the upper limit for HE to maintain a clear solution for higher light penetration. For instance, 1.51 equiv was used as the upper limit for HE in 2-propanol (IPA), given the solubility for HE in IPA was measured to be 60.47 mmol/L (Table S2). A clear reaction solution is important for achieving high and uniform light penetration to promote faster reactions and to avoid light scattering, which introduces an unknown parameter in the model, given the variations in particle size and quantity in different solvents, and for the algorithm to learn the relevant descriptors (i.e., intrinsic solvent properties). Lower and upper bounds of 0.5–5.0 mol % were selected for the Ir­(ppy)_3_ catalyst based on the UV–vis absorption spectra measurements (Figure S4), solubility in various solvents,^46^ and the range reported in the literature.^5,47^ Due to its catalytic role for reaction initiation and iminium ion (**Int-I**) formation, the propionic acid range was set as 0.1–1.0 equiv. The ranges for time and temperature were set based on preliminary data and reactor capability, respectively.

**Table 1:**
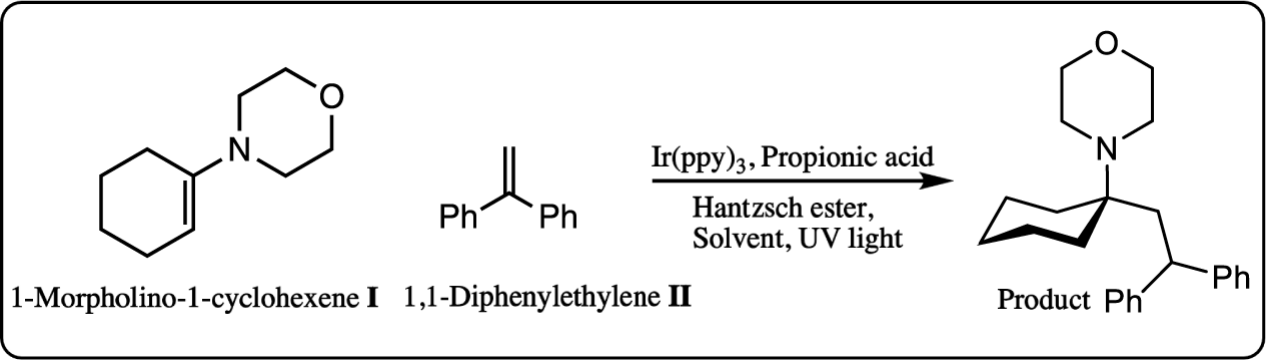
An Overview of the Reaction Scheme, Lower and Upper Bounds for Reaction Parameters, Solvent Descriptors, as Well as Solvent Selection Criteria

Variable	Lower bound	Upper bound
1,1-Diphenylethylene (**II**)/eq	1.0	2.0
Hantzsch ester/eq	1.0	2.0
*fac*-Ir(ppy)_3_/mol %	0.5	5.0
Propionic acid/eq	0.1	1.0
Temperature/°C	10	50
Residence time/min	4	60

Output	Metric
Cost	£ per experiment
Yield	%

Solvent descriptor	Meaning
Sig0	Surface area
Sig2	Electrostatic polarity
Sig3	Asymmetry of sigma profile
Hb_acc	Hydrogen bond acceptor strength
Hb_don	Hydrogen bond donor strength

Criteria	Example	Number of solvents
Liquid at 10 °C	Sulfolane	111
Basic	Pyridine	108
Water sensitive	Water	107
(Strongly) acidic	Triflic acid	101
UV cut-off limit	None	101
Solubility criteria	Methylformate	20

Starting with an initial list of 115 solvents, the discrete variable space was first reduced based on several criteria (e.g., removal of inherently basic solvents such as pyridine, given that the reaction is acid-catalyzed) as given in Table 1. HE and Ir­(ppy)_3_ catalyst solubilities were the most important criteria in selecting the candidate solvents. Even though suspensions can be pumped using the Vapourtec peristaltic pump, the presence of solids was found to block and scatter light. Therefore, solvents that can achieve a clear solution in a 40 mM reaction scale were selected based on the measured solubility values, with the full list provided in Table S8.

Five sigma moments were used for the parametrization of solvents due to their high information overlap with other sets, their time and cost efficiency due to being computationally calculated, and the ability to parametrize solvents in a small number of descriptors as opposed to sampling from a large descriptor space. Additionally, the usage of these sigma moments was rationalized by the work of Amar et al., who compared six models based on different descriptors for solvents in an asymmetric hydrogenation reaction, for objectives of conversion and diastereoselectivity.^28^ Compared to an information-rich model built on 17 descriptors, the authors found that the model built on five screening charge densities achieved higher accuracy in predicting low vs. high d.e., while model performances were comparable for predicting conversion. Moreover, Klamt and coworkers reported a comparison of experimental Abraham descriptors with the five computational COSMOments of Klamt’s COSMO-RS for 470 compounds.^35^ Using five sigma moments, the authors demonstrated a high information overlap between experimental Abraham descriptors and computational Klamt’s sigma moments.

Since each descriptor value and range are different, the descriptors were first standardized before implementing PCA. The solvent descriptors were reduced to two and three principal components, retaining 78.6% and 93.5% information, respectively (Figure S3). Moreover, the contributions of each of the original five descriptors to the new components are given in Figure S3. For example, the first component is highly dominated by electrostatic polarity, asymmetry of the sigma profile, and hydrogen bond acceptor strength. The second and third components are mainly defined by hydrogen bond donor strength and surface area, respectively, showing that all five components are included in the three-component space.

#### 3.1.2 UV–Vis and Photon Flux Studies

The Beer–Lambert law (eq 1) states that light penetration decreases exponentially over distance (*l*) for a given molar extinction coefficient (*e*) at a certain concentration (*c*), suggesting that reactions might take place only at the surface (of the reaction medium), leaving a significant area of “dark” zones inside the reaction medium that could lead to quenching of excited states or side reactions, especially if the reaction medium is limited by photon flux. Quantifying the influence of photon flux was demonstrated by Corcoran et al. when transferring optimal conditions to larger-scale reactors.^48^ Comparing 10, 60, and 150 mL reactors, the authors found that reaction yield versus absorbed photon equivalents followed the same line for all three reactors, suggesting that the reaction is driven by absorbed photon equivalents, not residence time alone. A similar study was reported by Lévesque et al. over lamp choices.^49^ For lamp intensities of 30, 60, and 82.5 W, product yield versus equivalents of emitted photons followed the same trajectory for three different lamps. Both studies highlight the importance of operating under conditions that are not limited by photon flux.

$$A=\log_{10}T=\log_{10}\frac{I_{0}}{I}=\mathrm{e\cdot c\cdot l}$$ 1

In the present study, three LED lamps from Vapourtec (365, 420, and 470 nm) were tested. Based on preliminary data, the highest yield was achieved with a 470 nm lamp under fixed conditions. Based on the UV–vis absorption spectra (Figure S4) measured from 200 to 800 nm, the *fac*-Ir­(ppy)_3_ catalyst absorbs under all three lamps, with a measured excitation coefficient of 1413.5 (mol/L)^−1^ cm^–1^ at 470 nm. Although both reactants absorb around 310–320 nm and hence do not affect light penetration, HE absorbs at 425 nm, affecting light penetration under the LEDs with wavelengths of 365 and 420 nm. Moreover, using lower-energy lamps could avoid potential side reactions, as it is less likely that other reaction species will absorb at longer wavelengths. Combining the preliminary data with the UV–vis absorption spectra, a 470 nm LED lamp was used for the optimization. Under irradiation with a 470 nm lamp, 1 mmol/L photocatalyst in 1 mm ID tubing results in an absorbance of 0.14. This allows for theoretical scalability to 10 mm ID (i.e., 100× volume increase from a 10 mL reactor to a 1 L scale) or an increase of catalyst concentration to 14 mM (Abs = 2) while maintaining a clear solution without sacrificing uniform light absorption.

A model to calculate the amount of photons radiated into the reaction medium is important to choose the appropriate reactor type and light source in accordance with the objectives (e.g., space-time yield maximization) the reaction is evaluated for.^50–52^ Photon flux received in the Vapourtec UV-150 photochemical reactor was studied using a potassium ferrioxalate actinometer (Figure S5). A model was developed to calculate absorption depending on the reactor depth, light source, wavelength, intensity, and irradiation (residence) time, based on the actinometer’s (potassium ferrioxalate) conversion to ferrous ion (Fe^2+^). Following the ferrous ion calibration (Figure S6) and the model equation (eqs S6 and S7), the standard 1 mm ID Vapourtec tubing in flow resulted in 30.9× more photon flux (Einstein s^–1^) or a 6.18× actinometric intensity of absorbed photons (Einstein s^–1^ L^–1^) over a Kessil Blue lamp in batch (4 mL vial), as summarized in Table S3. Experimental procedures, measurement techniques, and equations are reported in detail in the Supporting Information.

#### 3.1.3 Solubility Predictions Using COSMO*therm* and Measurements Using Benchtop NMR

Previous sections highlight the importance of working in a transparent region to optimize photon flux in the reaction medium. Running reactions as a suspension, though manageable in terms of hardware, significantly reduces light penetration and makes it difficult to quantify light scattering, thus increasing the risk of irreproducibility. For a reductive deiodination reaction, Nguyen et al. reported a 750% scaling of the reaction while simultaneously decreasing *fac*-Ir­(ppy)_3_ catalyst loading by 2000% and HE equivalents from 2.0 to 1.1 equiv without a significant loss in reaction efficiency. While attributed to serendipity, this is likely due to the presence of *fac*-Ir­(ppy)_3_ catalyst and HE in solid forms in the reaction. On the smaller scale, the catalyst concentration in the reaction corresponded to 2.5 mM, while it was only 0.38 mmol/L on the larger scale. Considering the measured solubility of 0.41 mmol/L for Ir­(ppy)_3_ in acetonitrile,^46^ the authors conducted the larger-scale reaction under fully transparent conditions, promoting higher light penetration. Therefore, in our study, experimental solubility results reported by Jespersen et al. were used to decide the upper limit for *fac*-Ir­(ppy)_3_ (Table S5) in case algorithm suggested values were above the solubility limit.^46^

Solubility values for HE were predicted using COSMO*therm* for the 107 solvents remaining from the original list.^53^ The software requires a reference list for solvents based on experimental measurements. Measuring solubility can be done via a solvent drying method, which works well with volatile solvents. For this reason, measuring concentrations with benchtop ^1^H NMR, with mesitylene as an internal standard, was also used for solubility measurements. Both approaches led to similar results, confirming the validity of either approach for the generation of solubility data (Table S8). COSMO*therm* solubility prediction accuracies were compared using 9, 18, and 35 references. While prediction accuracy improved with more experimentally measured references, predicted values using COSMO*therm* were used as a qualitative guide to select the solvents to be verified experimentally. We should note that using values of solubilities predicted by COSMO*therm* in this way should be done with the understanding that both underestimations and overestimations of solubilities may be within the predicted data. Thus, reviewing solvent classes that are selected based on COSMO*therm* predictions may be a useful step in ensuring no useful solvents were missed. Using a 40 mmol/L reaction concentration as a criterion and based on the measured solubility values, 20 solvents were selected to be used in the final solvent candidates list for optimization. For certain solvents, the solubility limit was used as the upper bound in running the reaction to achieve a clear solution. The procedure for both measurement approaches (Figure S7), accuracy comparisons (Table S4), and full solubility data (Table S8), both predicted and measured, are provided in the Supporting Information.

### 3.2 Training Data Set Collection and Algorithm-Guided Optimization

Latin hypercube sampling (LHS) was implemented separately in the three-component space to obtain eight solvents to generate the training data set for the NEMO algorithm. The objective of the training data set was to provide as much information about the reaction to the algorithm as possible. This is why several iterations of sampling were repeated to then decide on the best set of solvents; see Table 2. Similarly, LHS was implemented on six continuous variables with lower and upper bounds (Table 1) as a range from which to be sampled from. Six conditions per solvent were selected and used for the generation of training data. The list of solvents used in the training data set with their respective conditions for the highest and lowest yield in each solvent is given in Table 2.

**Table 2:** Part of the Training Data Set with the Highest and the Lowest Values for Yield in Each Solvent^a^

Entry	Alkene eq	Ir(ppy)_3_ mol %	HE eq	Acid eq	T/°C	Time/min	Solvent	Cost/£	Yield/%
1	1.81	1.14	1.56	0.27	28	68	DMF	1.40	26
2	1.19	2.33	1.81	0.72	43	5.5	DMF	2.05	5
3	1.81	1.14	1.56	0.27	28	68	DMSO	1.96	8
4	1.19	2.33	1.81	0.72	43	5.5	DMSO	2.62	2
5	1.81	1.14	1.24	0.27	28	68	Acetone	1.25	3
6	1.31	1.50	1.24	0.94	38	38	Acetone	1.42	0
7	1.69	2.92	1.44	0.49	35	113	NMP	2.38	19
8	1.19	2.33	1.60	0.72	43	5.5	NMP	2.09	7
9	1.70	3.05	1.44	0.50	33	25	DCM	2.19	71
10	1.06	1.45	1.07	0.60	48	53	DCM	1.25	10
11	1.69	2.92	1.35	0.49	35	113	Cyclohexanone	2.08	0
12	1.19	2.33	1.35	0.72	43	5.5	Cyclohexanone	1.75	0
13	1.56	3.05	1.19	0.38	18	98	THFA	2.19	0
14	1.44	0.55	1.28	0.83	23	23	THFA	0.89	0
15	1.94	0.85	1.94	0.16	13	83	EA	1.17	0
16	1.56	0.85	1.19	0.38	18	98	EA	1.01	0

a DMF: *N*,*N*-dimethylformamide, DMSO: dimethyl sulfoxide, NMP: 1-methyl-2-pyrrolidinone, DCM: dichloromethane, THFA: tetrahydrofurfuryl alcohol, EA: ethyl acetate.

NEMO was subsequently trained on the data set to suggest five conditions per iteration based on their highest combined EHVI. In terms of describing solvents, both the five sigma moments and the three principal components generated by applying PCA on the five sigma moments were benchmarked. XGBoost was selected as the best model for yield using three principal components, with a test RMSE of 3.66% and a test *R* ^2^ of 0.77. When using five sigma moments, NGBoost was selected as the best model for yield, with a model performance of test RMSE = 3.03% and test *R* ^2^ of 0.84. Therefore, optimization was conducted using six continuous variables and five sigma moments to describe solvents.

Conditions suggested by NEMO (orange points, Figure 3a) during the optimization included combinations of new solvents (e.g., acetonitrile), low-yield conditions, existing solvents (e.g., DCM), and high-yield conditions, suggesting a balance between exploration and exploitation. For purely exploitative algorithms, a selected model could have suggested conditions near the best result (e.g., 71% yield) provided in the training data (Table 2, row = 9). Over a few iterations, the algorithm suggested conditions focused on and around the experimentally identified Pareto front. After five iterations (25 data points), NEMO-suggested conditions had five points on the experimentally identified Pareto front versus three points found in the training data set of 48 points, highlighting the efficiency of NEMO to identify and populate the Pareto front.

**Figure 3:**
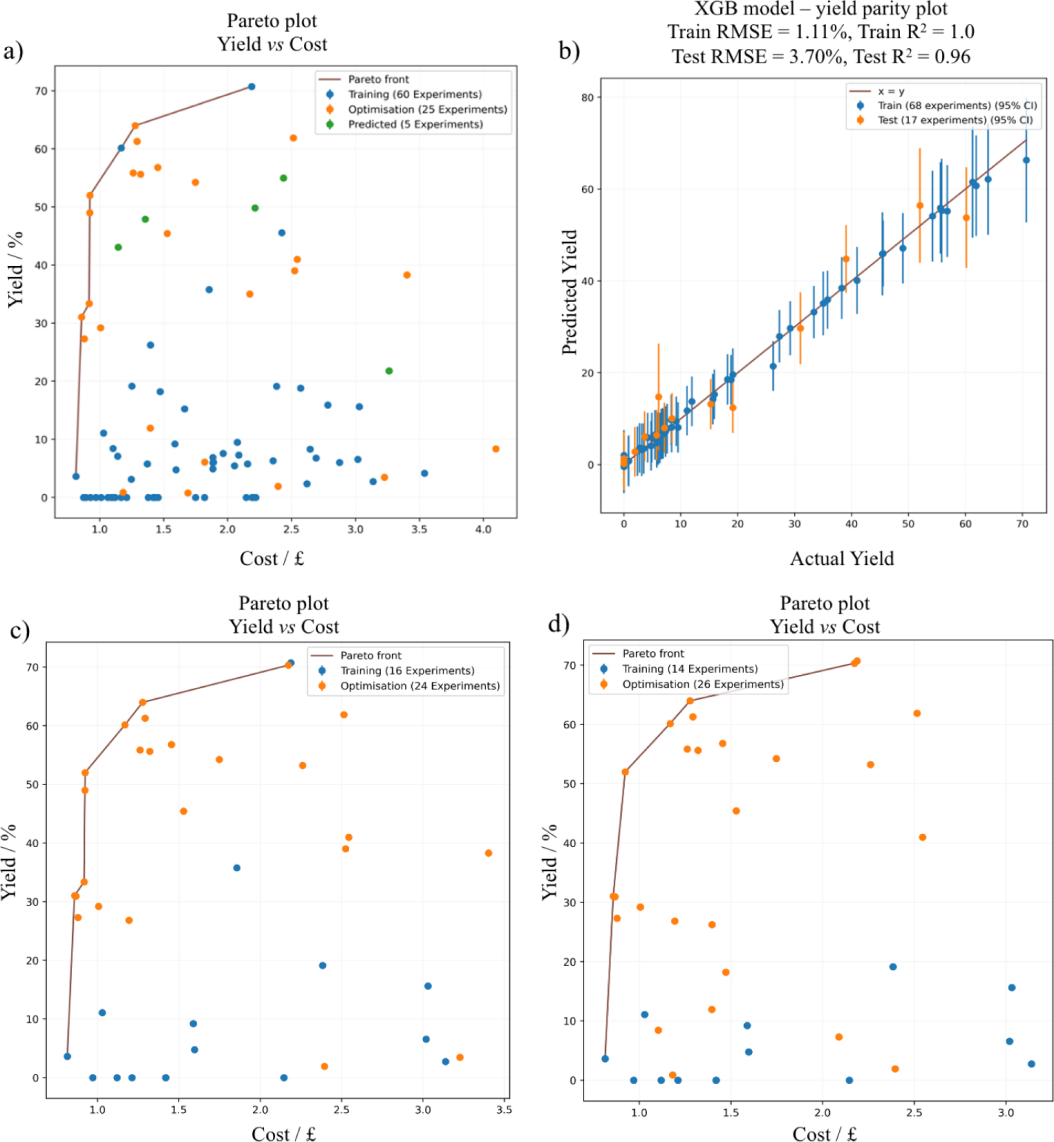
Algorithmic optimization of the reactions. (a) Optimisation plot (yield vs. cost) and Pareto front population using NEMO. (b) Parity plot for actual vs. predicted yield using the best model. Pool-based sampling and benchmarking NEMO with (c) 16 and (d) 14 training data points.

In terms of the stopping criteria, three conditions were used: Pareto front population, hypervolume improvement, and model predictive accuracy. When the optimization was initiated with 48 data points, the initial learning curve was steepest at the start, suggesting that new conditions were sampled based on the highest EHVI (Figure S8a). Over five iterations, hypervolume improvement converged, suggesting that the model has minimized uncertainties in unknown areas and achieved high prediction accuracy. This was also validated with test RMSE values when an 80/20 split was implemented with 5-fold cross-validation (CV). The RMSE for predicted values for yield was 3.7%, a low number considering the 1.80% experimental error from HPLC analysis (Figure S2), and the *R* ^2^ score was 0.96. Moreover, visual analysis of the Pareto front confirmed that most of the NEMO-suggested conditions were on or around the Pareto front, compared to low-yield and high-cost conditions in the training data set. All three conditions implemented as a stopping criterion confirmed that optimal conditions were found with a highly predictive model after balancing exploration and exploitation and the Pareto front to find trade-offs between the objectives of reaction yield and cost. Full data generated during the optimization is given in Table S6.

### 3.3 Benchmarking NEMO Using Pool-Based Sampling

To benchmark the learning efficiency of NEMO, the algorithm’s performance was evaluated using different data set sizes generated from the reaction optimization campaign. During the sampling, NEMO was forced to sample one condition per iteration from the pool of experimentally validated data sets based on the highest EHVI. In the first benchmark, NEMO was trained on 16 data points, two conditions per each of the eight solvents (Figure 3c). The training data set included a point on the Pareto front (Table 2, row 9). During the optimization, most of the points sampled by NEMO (orange points) were on or around the Pareto front, highlighting the learning efficiency of NEMO. This was reflected on the hypervolume learning over the experiment number (Figure S8b). After 20+ sampled points, hypervolume improvement had converged, suggesting that NEMO maximized the hypervolume learning. In the second benchmark, NEMO was trained using 14 data points, two points per each of the seven solvents, excluding DCM to avoid the presence of any points on the Pareto front (Figure 3d). Regardless, most of the sampled points were on or around the Pareto front, and the hypervolume improvement was maximized in 20+ sampled points. Using small data set sizes in each case, both with or without Pareto front points in the training data set, NEMO was able to maximize the hypervolume learning and populate the Pareto in a total of ∼40 experiments.

### 3.4 Explainable AI

Opening black-box models to extract insights and learn or validate physical knowledge about the reaction has gathered attention in the concept of explainable AI, to bridge the gap between physical models and black-box models.^54,55^ We implemented two approaches—partial dependence plot (PDP) and permutation feature importance (PFI)—to analyze the sensitivity of the objectives (yield and cost) to continuous variables and solvent descriptors. In PFI, the importance of a feature is calculated based on the model score when that feature is randomly shuffled.^56^ PDP, on the other hand, shows whether the relationship between a feature or two features and the target is linear, monotonic, or more complex.^57^ Both approaches identified sig3 (asymmetry of the s-profile) as the most important variable for yield (Figure 4). Lower values of sig3 corresponded to higher yield, identifying dichloromethane (DCM) and 1,2-dichloroethane (DCE) as the best solvents among 20 candidates included in the optimization. Solvent molecular area was selected as the second most important descriptor for solvents based on permutation feature importance. In terms of continuous variables, HE and 1,1-diphenylethylene **II** equivalency had the highest feature importance. This was followed by residence time and *fac*-Ir­(ppy)_3_ catalyst loading. It is important to note that correlated features could have low PFI or PDP values since the influence of one parameter could be masked by the other. For instance, a higher yield can be achieved at low catalyst loading with a longer residence time, since the reaction is driven by absorbed photon equivalence, which is the product of residence time and dissolved catalyst quantity. Acid equivalence and temperature had small influences on yield, with higher values correlated with higher yield. Cost is an analytical function and was influenced most by catalyst loading the most, followed by individual solvent cost. Partial dependence plots for all individual variables are provided in Figures S9 and S10.

**Figure 4:**
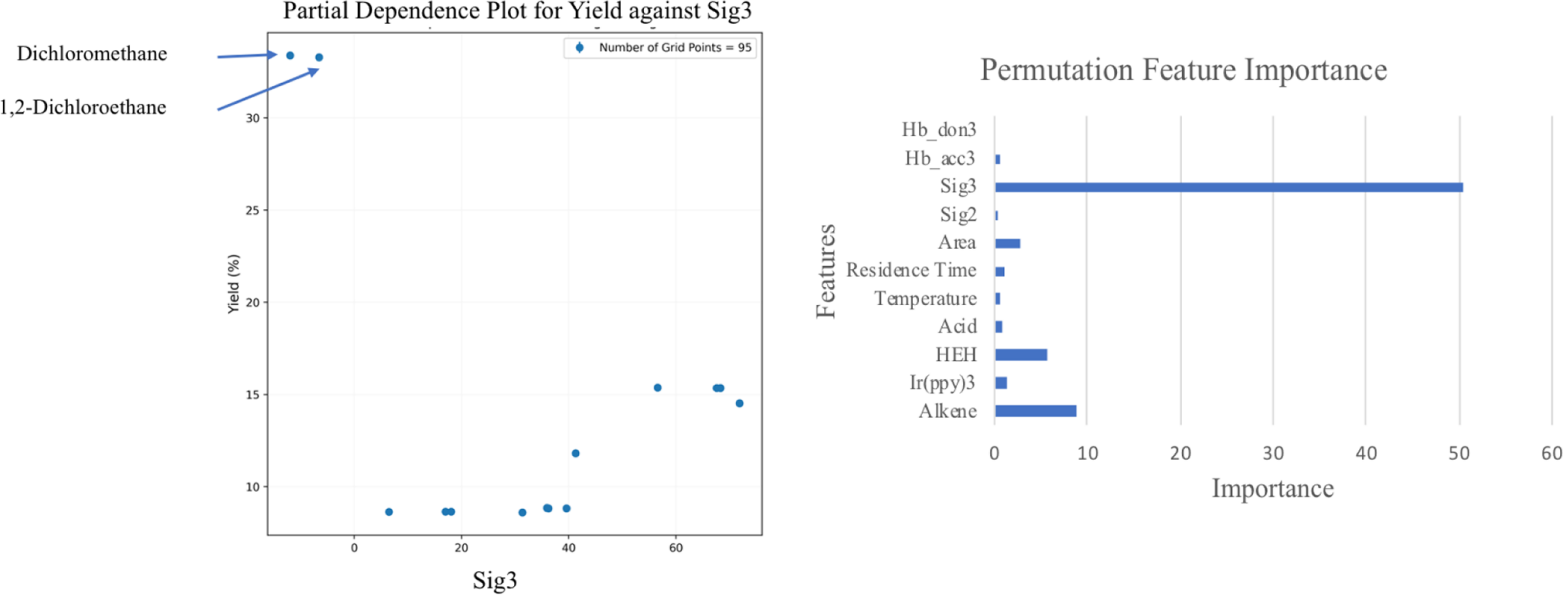
Partial dependence plot for Sig3, asymmetry of s-profile, against yield (left), and permutation feature importance of all variables for yield (right).

## 4 Conclusions

A workflow to generate *a priori* knowledge and develop a robust process in a semiautomated manner using Bayesian optimization has been demonstrated for a photoredox amine synthesis. Starting with 115 solvent candidates for discrete variables, the solvent candidates were shortlisted to 20 based on multiple screening criteria. UV–vis and photon flux studies provided a guideline for optimal reactor, lamp, and tubing selection. Solubility predictions were analyzed using the COSMO*therm* software. While quantitative values of the predictions were not used in the optimization, qualitative ranking of solvents based on the predicted solubility values served as a guideline to experimentally validate solubility values for HE using a benchtop NMR. The recently developed NEMO algorithm was used to build a robust model, using five black-box surrogate models and sampling based on the highest EHVI for combinations of suggested conditions. A Pareto front was identified for two objectives: reaction yield and cost, and it was populated based on the recommended conditions by NEMO. While the reaction was not directly optimized for productivity, the highest productivity value achieved equals to ∼12 g/day scale-up using a Vapourtec UV-150 reactor and was ∼25× higher than the optimal batch result, still leaving room for the theoretical scale-up of the reactor from 10 mL to 1 L without sacrificing light penetration. The development of this case study illustrates a general approach for development in process chemistry, which constrains experimental space potentially accessible to robotic self-optimization systems by identifying feasible conditions through the assembly of reaction context, preparative knowledge, the viable limits of concentrations, and the interactions between process inputs such as light intensity and solvent choice. While the generation of prior knowledge in this study is manual, we are further developing the technology for automated identification of the relevant process context.

## Supplementary Material



## Notes

Experimental details, solvent properties, calibrations, and solubility studies (PDF)
