# Supplementary material for: Machine Learning-Driven Optimization of Continuous-Flow Photoredox Amine Synthesis
Source: Org Process Res Dev. 2025 May 21;29(6):1411–22. doi: 10.1021/acs.oprd.4c00533 (PMC12186674; doi:10.1021/acs.oprd.4c00533)
Supplement: Supplementary file 1 [file op4c00533_si_001.pdf]

Electronic Supplementary Information (ESI)

for

Machine learning-driven optimisation of continuous flow photoredox amine synthesis

Perman Jorayev,[1,3] Sebastian Soritz,[1,2] Simon Sung,[3] Mohammed I. Jeraal,[3] Danilo Russo,[1,4] Alexandre Barthelme,[5] Frédéric C. Toussaint,[5] Matthew J. Gaunt[6] and Alexei A. Lapkin*[1,3]

[1] *Department of Chemical Engineering and Biotechnology, University of Cambridge, Cambridge CB3 0AS, United Kingdom*

[2] *Astex Pharmaceuticals, 436 Science Park, Cambridge, CB4 0QA, United Kingdom*

[3] *Cambridge Centre for Advanced Research and Education in Singapore, CARES Ltd. 1 CREATE Way, CREATE Tower #05-05, Singapore 138602, Singapore*

[4] *Department of Chemical Engineering, Materials, and Industrial Production, University of Naples Federico II. Piazzale V. Tecchio 80, 80125, Naples, Italy*

[5] *UCB Pharma S.A. Allée de la Recherche, 60 1070 Brussels, Belgium*

[6] *Yusuf Hamied Department of Chemistry, University of Cambridge CB2 1EW, United Kingdom*

Table of Contents

Reaction chemistry and experimental setup……………………………………...…………2

HPLC calibration for 4-(1-(2,2-diphenylethyl)cyclohexyl)morpholine……………………….3

Sigma moments and PCA analysis for solvents……………………………………………….3

List of solvents and respective sigma moments calculated using COSMOtherm……………..4

List of selected solvents and the associated cost………………………………….…………...5

UV-Vis absorption of reaction components……………………………………….............6

Photon flux study……………………………………………………………………………...7

Calibration line for Fe^{+2} complex formation with 1,10-phenanthroline………………………8

Comparison of photon flux received in batch *vs* flow………………………………………10

Experimental solubility measurements of Hantzsch ester in various solvents……………......11

* Corresponding Author; A. Lapkin. Email: aal35@cam.ac.uk

Maximum solubility of the catalyst *fac*-Ir(ppy)$_3$ in commonly used solvents ………….......12
NEMO benchmarking using pool-based sampling……………………………………………..13
Explainable AI results………………………………………………………………………...14
All the data generated during the training and optimisation……………………………….....16
Predicted and measured Hantzsch ester solubility in various solvents……………………......18
References……………….…………………………………………………………………...21

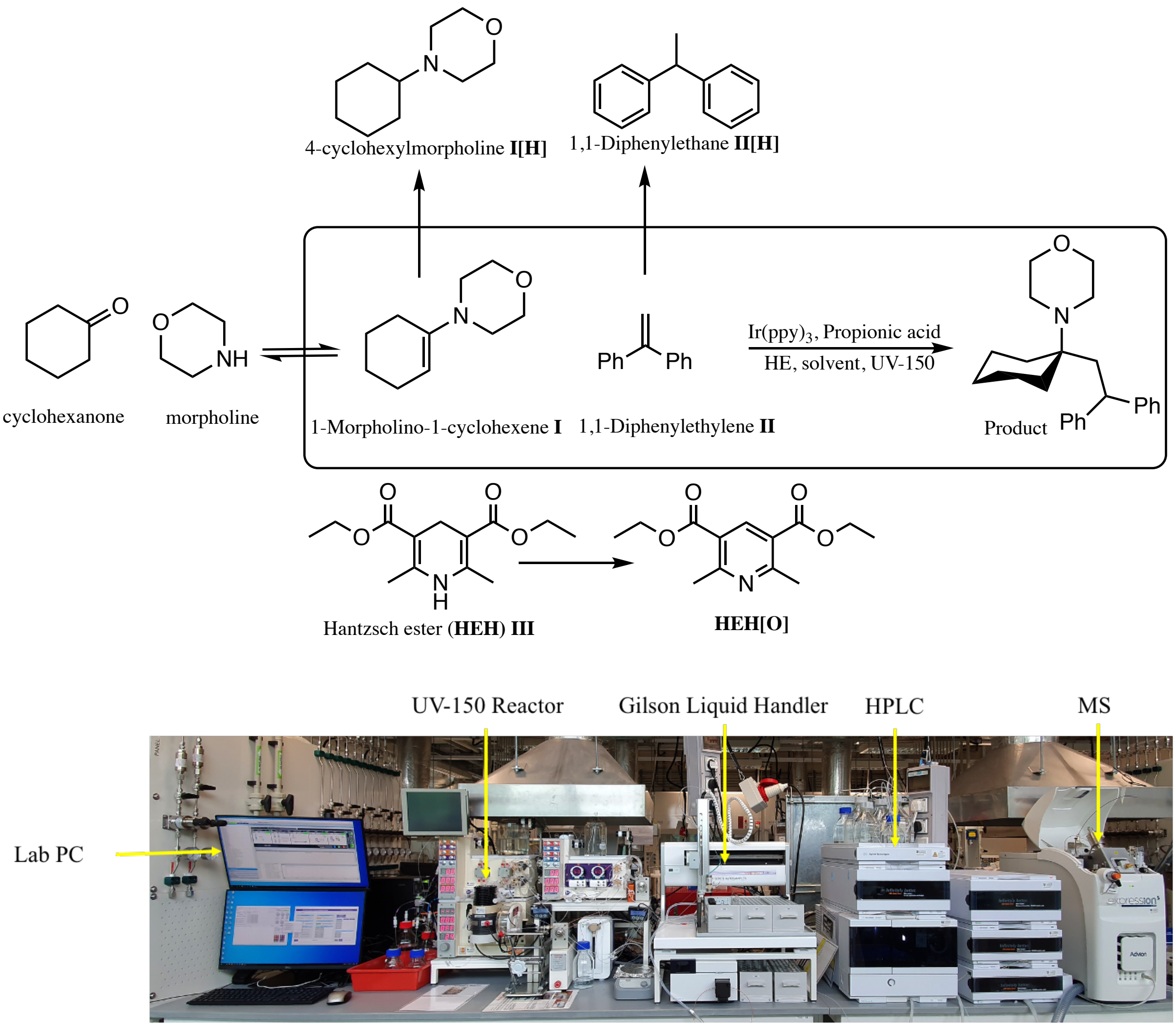

***Figure S1**. Reaction chemistry and experimental setup for photoredox amine synthesis with identified competing side reactions.*

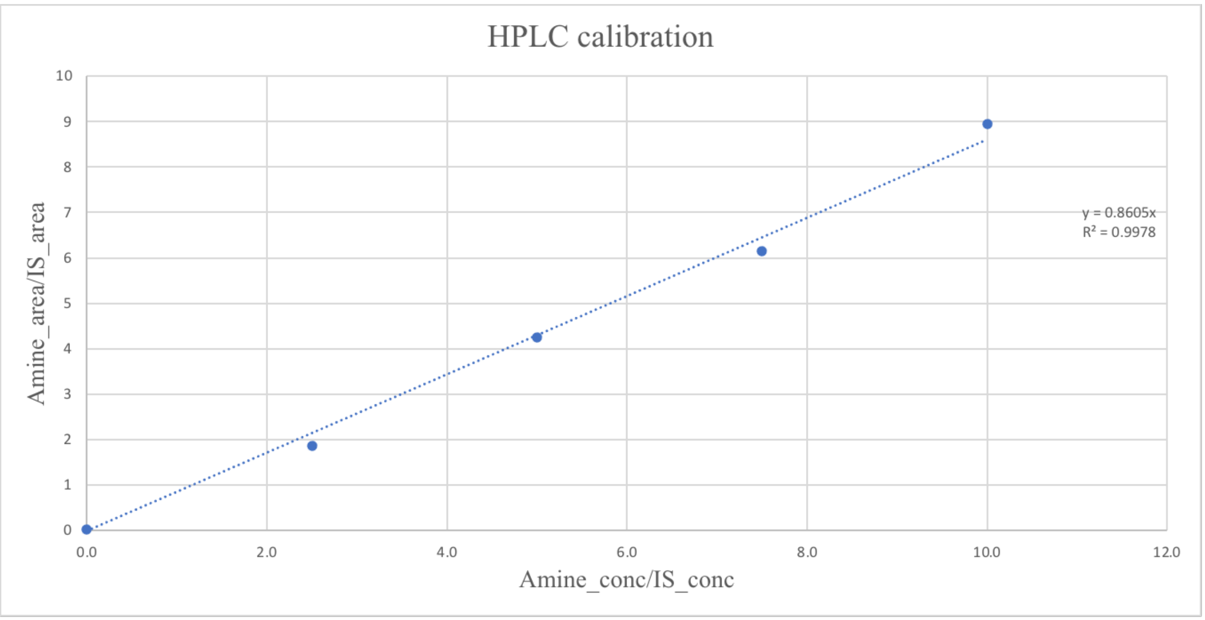

Figure S2. *HPLC calibration for amine product (**III**) vs mesitylene internal standard. Standard deviation for yield based on analysing the same vial three times was 1.80%.*

Component	Information / %	Dimensions	Information / %
PC1	47.7	2D	78.6
PC2	30.9	3D	93.5
PC3	14.9		

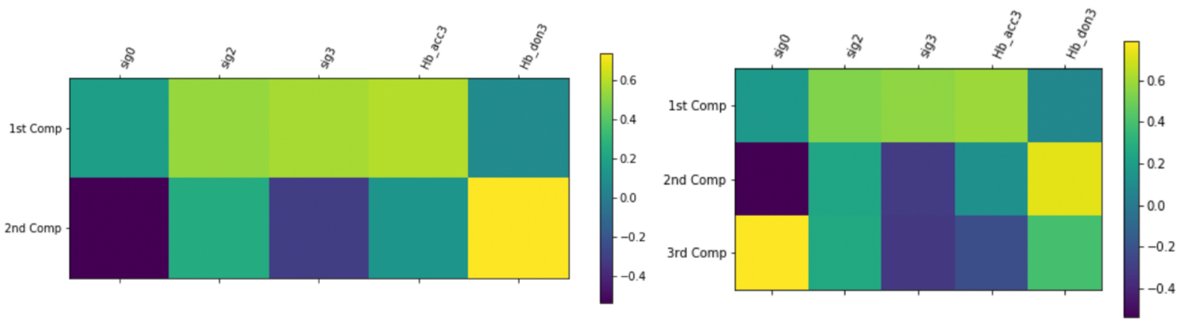

Figure S3. *Information percentage retained after implementing PCA on five sigma moments (top). Contribution of each of the initial descriptors to the principal components (bottom).*

***Table S1**. List of solvent candidates used in optimisation and respective sigma moments calculated using COSMOtherm.*

Solvent No	Molecule	Abbv	Area	sig2	sig3	Hb_acc3	Hb_don3
1	1,2-dichloroethane	DCE	118.7231	38.945	-6.58605	0	0
2	1,3-dimethyl-2-imidazolidinone	DMI	154.8425	64.9879	63.6522	3.6463	0
3	1,3-dimethyltetrahydropyrimidin-2(1h)-one	DMPU	168.3184	62.7928	67.5844	4.4743	0
4	2-furanmethanol	Furufyrl alcohol	134.4065	66.98897	6.457333	2.112433	1.598867
5	2-methyltetrahydrofuran	2-MeTHF	132.8992	31.94015	30.65855	2.60355	0
6	2-propanol	IPA	108.1837	46.9444	21.06115	2.86455	1.1897
7	acetonitrile	MeCN	83.3227	48.4098	16.9976	0.901	0
8	benzylalcohol	BnOH	151.4705	60.32833	6.469025	2.006225	1.572775
9	dimethylformamide	DMF	117.0929	60.0196	56.594	3.8646	0
10	dimethylsulfoxide	DMSO	112.8855	78.6819	71.8756	4.7426	0
11	cyclohexanone		140.5918	46.7592	41.2752	2.8451	0
12	ethanol	EtOH	89.36075	46.6555	18.08005	2.7002	1.3905
13	ethylacetate	EA	134.4695	54.34033	36.22283	2.136233	0
14	n,n-dimethylacetamide	DMA	134.0875	61.5682	63.605	4.3094	0
15	n-methyl-2-pyrrolidinone	NMP	140.6449	63.1739	68.2801	4.3578	0
16	propanone		103.3383	47.4029	35.9321	2.6007	0
17	tetrahydrofurfurylalcohol	THFA	141.433	65.53496	39.59406	4.34038	1.14161
18	THF	THF	113.6072	31.6911	31.345	2.6497	0
19	CH2Cl2	DCM	99.3069	28.9636	-11.9892	0	0.1088
20	dimethylisosorbide		201.5833	86.60345	65.88202	5.075417	0

***Table S2**. List of final solvents used during the optimisation and the associated cost.*

Solvent No	Molecule	Abbv.	Hantzsch ester pred. solublity / mM	Hantzsch measured solubility / mM	Price £ / L	Price £ / 5 mL (rxn vol)	Purchase vol / L
1	1,2-dichloroethane	DCE	27.91	38.06	69.5	0.3475	2
2	1,3-dimethyl-2-imidazolidinone	DMI	91.88	309.25	648	3.24	0.5
3	1,3-dimethyltetrahydropyrimidin-2(1h)-one	DMPU	156.43	357.02	370	1.85	1
4	2-furanmethanol	Furufyrl alcohol	36.82	86.45	99.9935	0.499968	1
5	2-methyltetrahydrofuran	2-MeTHF	39.57	122.21	141	0.705	2
6	2-propanol	IPA	16.98	60.47	44.4	0.222	2
7	acetonitrile	MeCN	11.25	19	99	0.495	2
8	benzylalcohol	BnOH	42.04	91.2	203	1.015	2
9	dimethylformamide	DMF	99.22	178.33	84.5	0.4225	2
10	dimethylsulfoxide	DMSO	30.98	159.51	198	0.99	2
11	cyclohexanone		41.51	-	41.4	0.207	1
12	ethanol	EtOH	30.28	45.02	80	0.4	2
13	ethylacetate	EA	40.79	80.56	53.5	0.2675	2
14	n,n-dimethylacetamide	DMA	158.03	330.22	75.5	0.3775	2
15	n-methyl-2-pyrrolidinone	NMP	172	390.82	99.5	0.4975	2
16	propanone		31.65	49.71	66.8	0.334	1
17	tetrahydrofurfurylalcohol	THFA	36.74	-	58.0754	0.290377	1
18	THF	THF	84.82	93.96	73.5	0.3675	2
19	CH2Cl2	DCM	118.66	64.11	46.25	0.23125	2
20	dimethylisosorbide		15.52	-	114.4	0.572	2.5

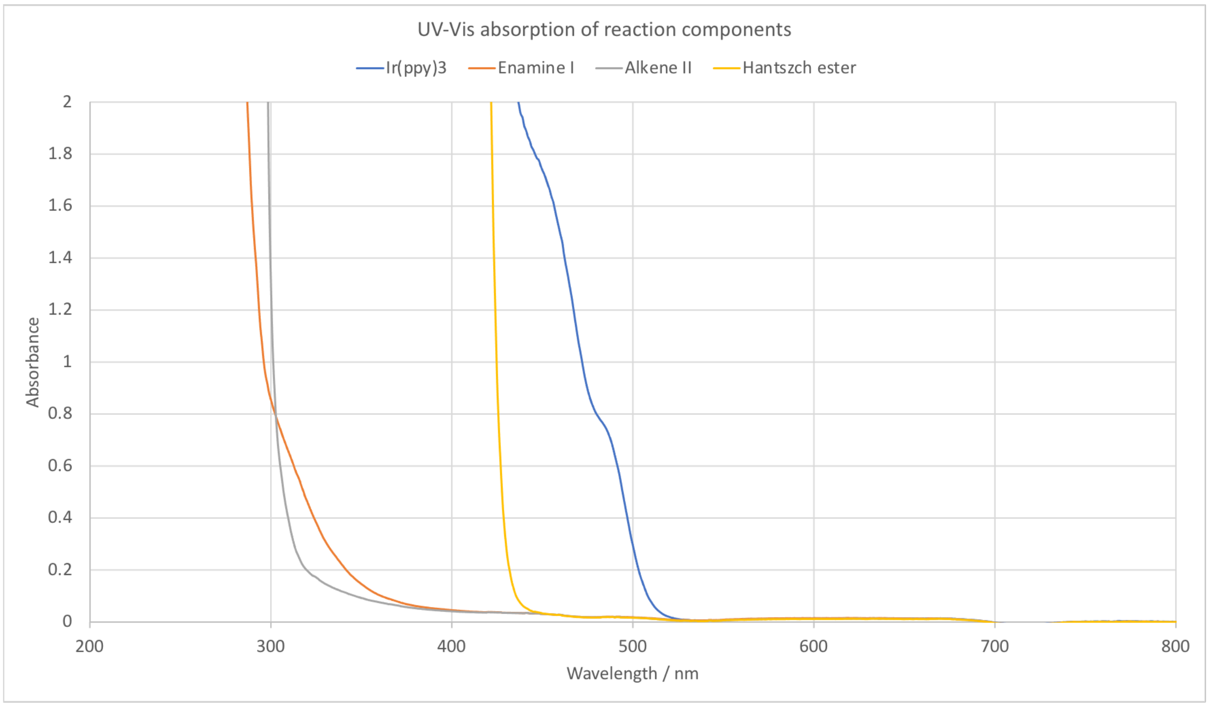

Figure S4. *UV-Vis absorption of reaction components.*

Photon flux study

Experimental

$$2FeCl_3 + 6K_2C_2O_4 \rightarrow 2\ K_3[Fe(C_2O_4)_3] + 6KCl$$

Potassium ferrioxalate was synthesised by mixing 1.5 mol L^{-1} $6K_2C_2O_4\ H_2O$ and 1.5 mol L^{-1} $FeCl_3$ at 3:1 volume ratio. The mixture was rigorously stirred to give potassium ferrioxalate precipitate, which was filtered, recrystallised three times with water, and dried overnight in oven at 40 °C. For actinometry studies in batch (4.0 mL glass vial) using Kessil Blue lamp and in flow using Vapourtec UV-150 reactor (10.0 mL), appropriate amounts of potassium ferrioxalate solutions (C_{A0}), specifically 0.006 mol L^{-1} (2.947 g) and 0.15 mol L^{-1} (73.68 g), were prepared in 0.05 mol L^{-1} H_2SO_4. For analysis, based on the amount of ferrous ion produced, 650 µL of irradiated solution was mixed with 26 µL of o-phenanthroline solution (0.05 mol L^{-1}), 1150 µL of CH_3COONa (1.0 mol L^{-1}), 750 µL of H_2SO_4 (0.5 mol L^{-1}), and analysed using UV-Vis. O-phenanthroline concentration was at least three times higher than Fe^{+2} concentration to ensure complex formation. Absorption coefficient of $Fe^{+2}(phen)_3$ complex at 512 nm was measured to be 9,918 L mol^{-1} cm^{-1}, compared to 10,910 L mol^{-1} cm^{-1} reported by Lehoczki *et al*. and 10,980 L mol^{-1} cm^{-1} (at 510 nm) reported by Aillet *et al*.

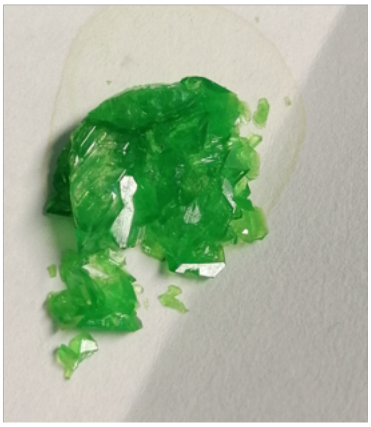

$K_3[Fe(C_2O_4)_3]\text{x}3\text{H}_2\text{O}$

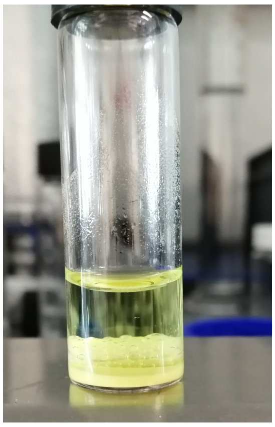

$K_3[Fe(C_2O_4)_3]\ precipite$

***Figure S5**. (left) potassium ferrioxalate crystal and (right) precipitate.*

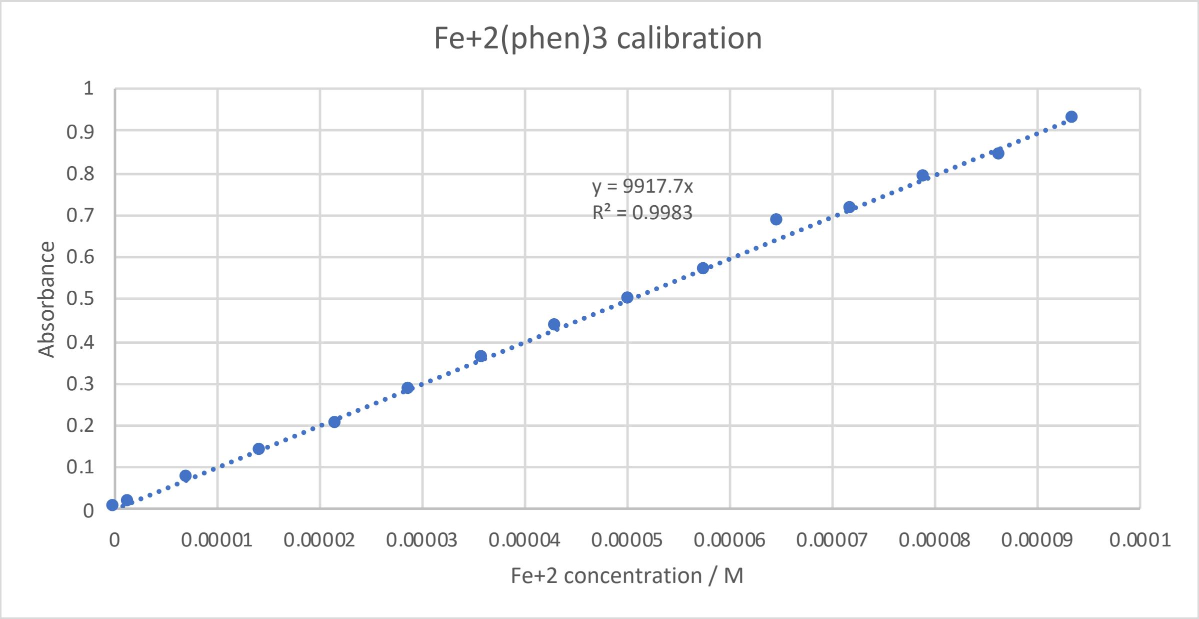

Figure S6. *Calibration line for Fe^{+2} complex formation with 1,10-phenanthroline at 512 nm.*

Modelling

Photodecomposition of potassium ferrioxalate in water is given as following.

$$2[Fe(C_2O_4)_3]^{3-} \rightarrow 2Fe(C_2O_4) + 3[C_2O_4]^{2-} + 2CO_2$$

Assuming a perfect mixing in the continuous flow reactor in Vapourtec, potassium ferrioxalate decomposition can be described as the following and the consumption rate of potassium ferrioxalate (A), and conversion to ferrous ion (B), is linearly related to the quantum yield (φ_λ / mol einstein^{-1}) of the reaction and the mean absorbed photon flux density ($< L^a_{p,\lambda} >$ / einstein m^{-3} s^{-1}) at wavelength λ nm (equation 3).

$$A \overset{hv}{\rightarrow} B \qquad (S1)$$

$$-\frac{dC_A}{dt} = \varphi_\gamma < L^A_{p,\lambda} > \qquad (S2)$$

$$\langle L^A_{p,\lambda} \rangle = \frac{q_{p,\gamma}}{V_r} f_\lambda \qquad (S3)$$

Where f_λ is the fraction of the absorbed light and $q_{p,\gamma}$ (einstein s^{-1}) is the photon flux received over the entire volume in a reactor V_r (m^3). The fraction of the absorbed light could be calculated based on the molar absorption coefficient κ_λ (m^2 mol^{-1}) of A in a given path length l (m) as following:

$$f_\lambda = 1 - e^{-A_e} = 1 - e^{-\kappa_\lambda C_A l} \quad (S4)$$

The received photon flux over the entire reactor volume depends on the reactor material transmittance (T_λ) and the incoming photon flux ($q_{p0,\lambda}$) at the surface, and can be expressed as

$$q_{p,\lambda} = q_{p0,\lambda}\, T_\lambda \quad (S5)$$

For fluoropolymer (FEP) reactor tubing of Vapourtec UV-150 reactor, material transmittance at 512 nm was taken as 1.0.

For monochromatic light source, reactor material transmittance (T_λ) is equal to 1.0 and is constant, which means $q_{p,\lambda} = q_{p0,\lambda}$. Moreover, both $q_{p,\gamma}$ and κ_λ are constant. Integrating equation 3 from C_{A0} to C_A, and $t = 0$ to $t = \tau$, based on equations 3-6 leads to integrated equation 7 to quantify the photon flux $q_{p,\gamma}$ (einstein s^{-1}) received over the absorbing volume.

$$\left(\varphi_\gamma \frac{q_{p,\gamma}}{V_r}\right)\tau = C_{A0}X + \frac{1}{\kappa_\lambda l}\ln\left[\frac{1 - e^{-\kappa_\lambda C_{A0} l}}{1 - e^{-\kappa_\lambda C_{A0}(1-X) l}}\right] \quad (S6)$$

where $C_A = C_{A0}(1 - X)$ for residence time $\tau = \frac{V_r}{Q}$.

For a polychromatic light source, the light wavelength λ is not constant, which means both $q_{p,\gamma}$ and κ_λ are not constant. The received photon flux needs to be integrated over discrete wavelengths intervals $\Delta\lambda_i$, together with the lamp density function g_λ at various wavelengths. As demonstrated by Aillet et al.[1] received photon flux for a polychromatic light source can be quantified as

$$\frac{dX}{dt} = \frac{1}{C_{A0}}\left(\frac{q_{p,0}}{V_r}\right)\sum\nolimits_{\Delta\lambda_i}\left[T_{\lambda_i}\varphi_{\lambda_i} g_{\lambda_i}\left(1 - e^{-\kappa_{\lambda_i} C_{A0}(1-X) l}\right)\right] \quad (S7)$$

where $q_{p,0}$ is the total incoming photon flux. For each wavelength λi and the power $M_{p,\lambda}$ emitted at that wavelength, the density function g_λ of the lamp can be express as following:

$$g_\lambda = \frac{M_{p,\lambda}}{\sum_{\lambda_i} M_{p,\lambda_i}} \quad (S8)$$

Results

Table S3. *Comparison of photon flux received in batch under Kessil blue lamp and in flow under 470 nm LED. The results are compared with different lamps and reactor setups reported by Aillet et al.*[1] *and Loponov et al.*[2]

**Reactor Type**	**Light type**	**Wavelength / nm**	**Lamp**	**Reactor volume / mL**	**Photon flux ($x10^6$) / Einstein s^{-1}**	**Actinometric intensity of absorbed photons ($x10^4$) / Einstein L^{-1} s^{-1}**
Aillet et al. microphotoreactor - flow	Polychromatic	-	High pressure Hg	0.81	26.2	323.46
Aillet et al. microphotoreactor - flow	Monochromatic	365	UV-LED	0.54	0.38	7.07
Aillet et al. immersion well	Polychromatic	-	High pressure Hg	225	47.60	2.16
This work – batch	Polychromatic	-	Kessil Blue lamp	4	0.20	1.00
This work - flow	Monochromatic	470	VT UV-150	10	6.18	6.18
Loponov et al.	Monochromatic	420	FL			5.7
	Monochromatic	524	LED			4.6
	Polychromatic	-	Xe arc			2.3
	Polychromatic	-	Hg MP (IW)			1.2

Solubility predictions and measurements

Experimentally reported solubility values for Ir(ppy)$_3$ catalyst for common solvents was used.[3] Solubility of Hantzsch ester was measured using direct solvent evaporation technique. First, a suspension of Hantzsch ester in a given solvent with pre-weighed vial was prepared and stirred for 1 h. The suspension was filtered to accommodate a clear solution (1 mL). The solvent was dried by blowing air over the solution. Final weight of the vial was measured to calculate the total dissolved amount of Hantzsch ester. Similarly, 1 mL clear solution was prepared to be analysed via benchtop NMR with mesitylene as an internal standard.

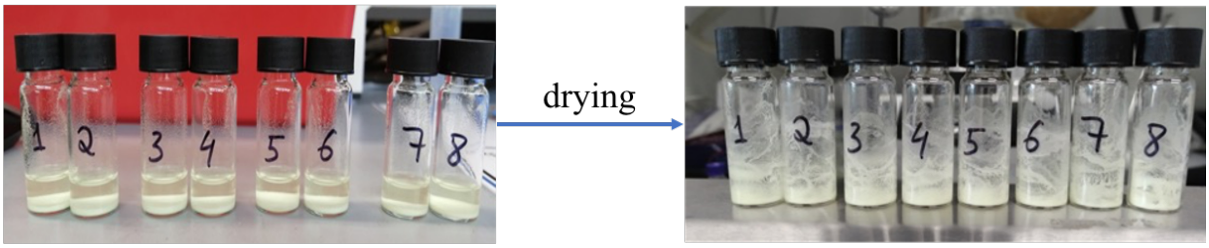

Figure S7*. Preparation of Hantzsch ester suspensions and drying process.*

Table S4*. Comparison of solubility results via air drying vs benchtop NMR analysis.*

Solvent	Solubility (mg /mL) - measured using solvent drying	Solubility (mg /mL) - measured using benchtop NMR
Methanol	5.60	5.60
Diethyl ether	1.00	1.00
Chloroform	36.00	38.54
Acetone	10.40	12.59
Hexane	0.01	0.01
Acetonitrile	4.40	4.81
Diethyl carbonate	2.80	4.50
Dichloromethane	15.00	16.24
N,N-dimethylformamide	52.80	45.17
1-butanol	6.80	5.66

Table S5. *Maximum solubility of the catalyst fac-Ir(ppy)$_3$ in commonly used solvents. Reproduced from ref.*[3]

Solvents	Molar concentration
Acetone	6.0x10^{-4}
Acetonitrile	4.1x10^{-4}
Dichloromethane	7.3x10^{-3}
N,N-Dimethylformamide	1.5x10^{-3}
Dimethylsulfoxide	3.7x10^{-3}
Ethyl Acetate	3.4x10^{-4}
Methanol	1.1x10^{-5}
Methyl-t-butyl ether	6.2x10^{-5}
N-Methyl 2-pyrrolidinone	5.2x10^{-2}
Tetrahydrofuran	2.1x10^{-3}
Toluene	5.9x10^{-4}

COSMOtherm for solubility predictions

Available solvents in the COSMO*therm* database of 1401 solvents were loaded automatically in the software with BP_TZVPD_FINE parameterisation. If a certain solvent was not available, conformer search and structure optimisation were performed using COSMO*conf* program. Depending on the run, experimental references values were for Hantzsch ester solubility in 9, 18, or 35 solvents were provided. Choices of solvents in the reference list were chosen to maximise the diversity of solvent classes.

Benchmarking results using pool-based sampling

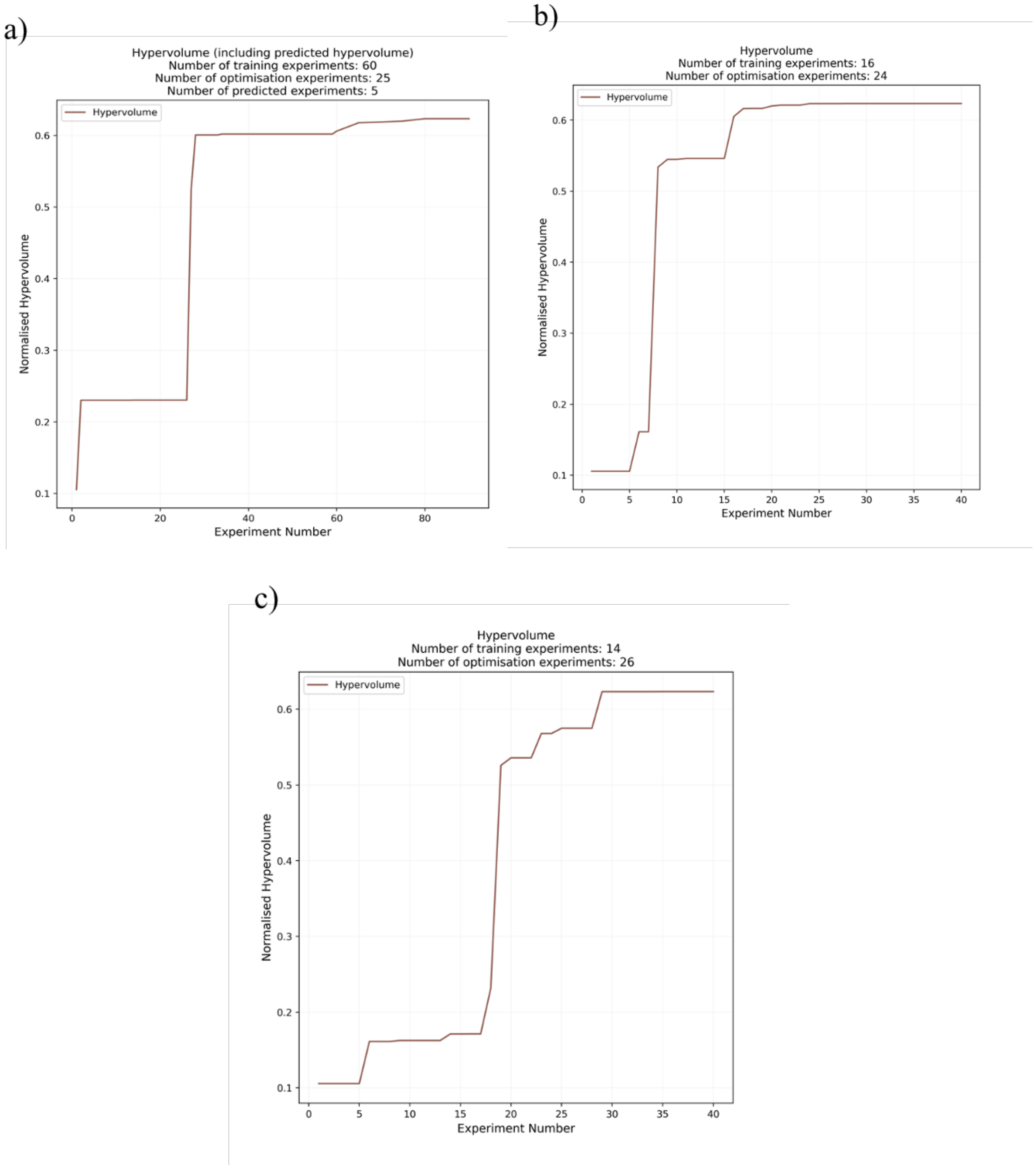

***Figure S8**. Hypervolume improvement over experiment number for a) full optimisation, b) benchmarking using 16 training points, including a point on the Pareto front, and c) 14 training points with no Pareto points included.*

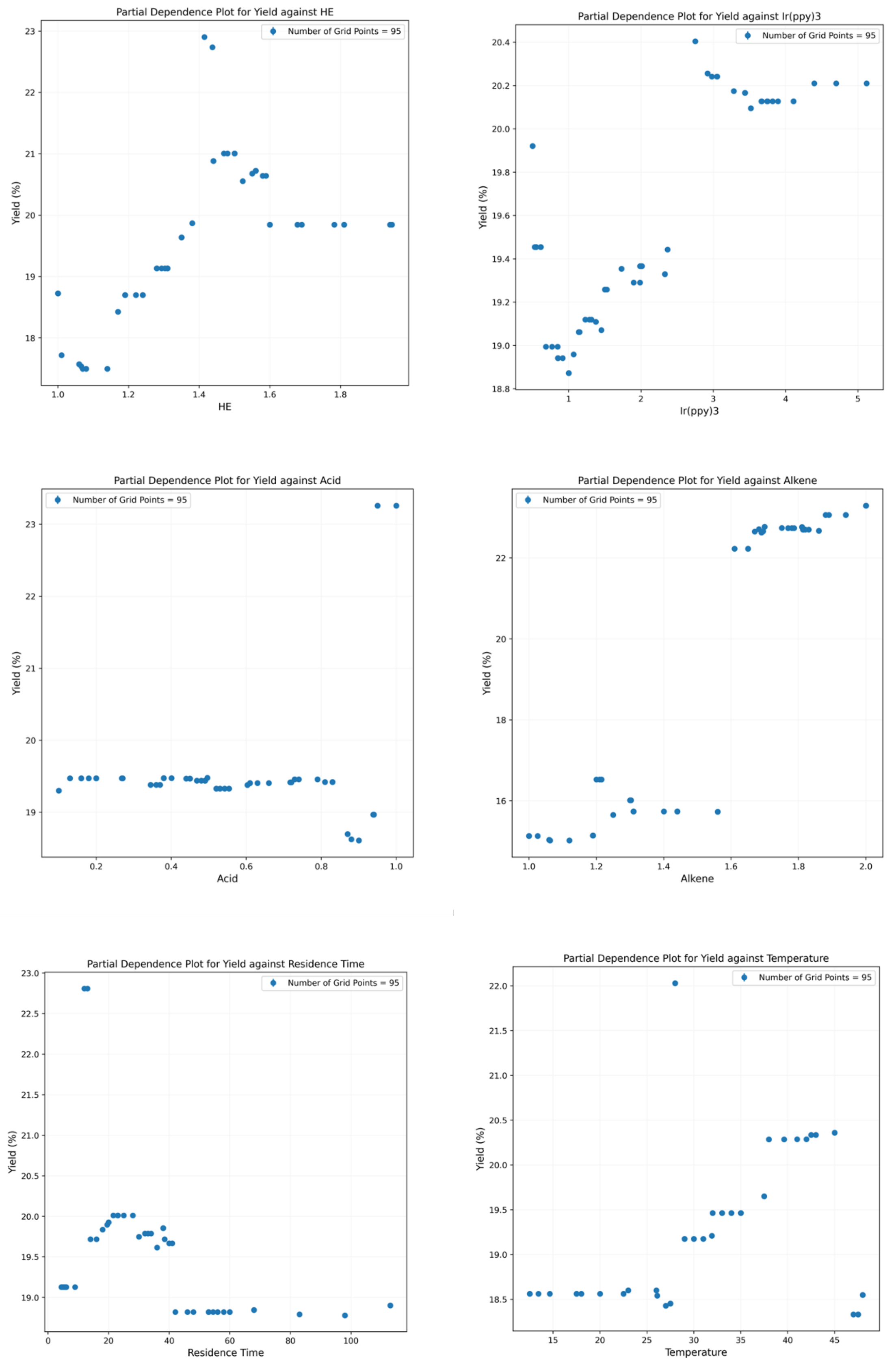

Figure S9. *Partial dependence plot for continuous variables against yield.*

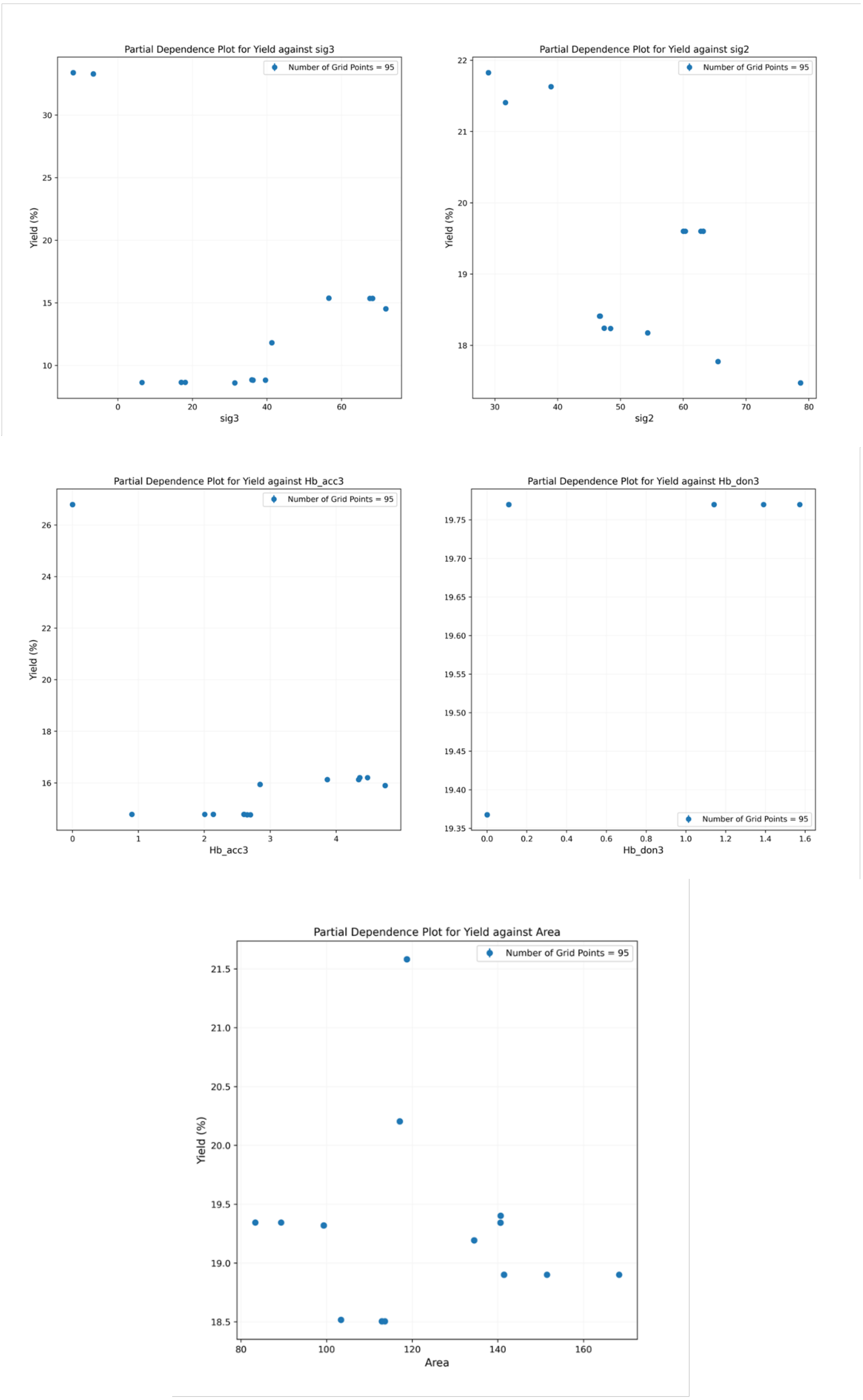

Figure S10. *Partial dependence plot for all solvent descriptors against yield.*

Table S6*. Data generated during the training and optimisation.*

Rxn No	Rxn type	Alkene eq.	Ir(ppy)$_3$ mol%	HEH eq.	Acid eq.	T / C	Time / min	Solvent	Cost / £	Yield / %
1	training	1.06	1.73	1.06	0.61	18	98	DMF	1.588	9.20
2	training	1.44	0.55	1.31	0.83	23	23	DMF	1.029	11.09
3	training	1.81	1.14	1.56	0.27	28	68	DMF	1.397	26.23
4	training	1.31	3.75	1.69	0.94	38	38	DMF	2.783	15.90
5	training	1.19	2.33	1.81	0.72	43	5.5	DMF	2.053	5.47
6	training	1.56	3.52	1.19	0.38	48	53	DMF	2.571	18.81
7	training	1.56	3.52	1.19	0.38	18	98	DMSO	3.138	2.76
8	training	1.44	0.55	1.31	0.83	23	23	DMSO	1.596	4.77
9	training	1.81	1.14	1.56	0.27	28	68	DMSO	1.964	7.58
10	training	1.69	2.92	1.44	0.49	35	113	DMSO	2.875	6.03
11	training	1.31	4.11	1.69	0.94	38	38	DMSO	3.540	4.18
12	training	1.19	2.33	1.81	0.72	43	5.5	DMSO	2.621	2.36
13	training	1.06	1.73	1.06	0.61	48	53	DMSO	2.155	5.79
14	training	1.56	1.50	1.19	0.38	18	98	Acetone	1.419	0.00
15	training	1.44	0.55	1.24	0.83	23	23	Acetone	0.927	0.00
16	training	1.81	1.14	1.24	0.27	28	68	Acetone	1.247	3.14
17	training	1.69	1.50	1.24	0.49	35	113	Acetone	1.433	0.00
18	training	1.31	1.50	1.24	0.94	38	38	Acetone	1.423	0.00
19	training	1.19	1.50	1.24	0.72	43	5.5	Acetone	1.418	0.00
20	training	1.06	1.50	1.06	0.61	48	53	Acetone	1.378	0.00
21	training	1.56	3.52	1.19	0.38	18	98	NMP	2.646	8.30
22	training	1.44	0.55	1.31	0.83	23	23	NMP	1.104	8.43
23	training	1.81	1.14	1.56	0.27	28	68	NMP	1.472	18.20
24	training	1.69	2.92	1.44	0.49	35	113	NMP	2.383	19.13
25	training	1.31	4.11	1.60	0.94	38	38	NMP	3.030	15.62
26	training	1.19	2.33	1.60	0.72	43	5.5	NMP	2.088	7.29
27	training	1.06	1.73	1.06	0.61	48	53	NMP	1.663	15.26
28	training	1.81	1.07	1.56	0.27	28	68	DCM	1.168	60.14
29	training	1.70	3.05	1.44	0.50	33	25	DCM	2.187	70.71
30	training	1.30	3.44	1.68	0.94	38	38	DCM	2.424	45.55
31	training	1.19	2.37	1.68	0.72	43	5.0	DCM	1.856	35.78
32	training	1.06	1.45	1.07	0.60	48	53	DCM	1.251	19.16
33	training	1.94	4.70	1.35	0.16	13	83	Cyclohexanone	3.019	6.57
34	training	1.56	3.52	1.19	0.38	18	98	Cyclohexanone	2.355	6.31
35	training	1.44	0.55	1.31	0.83	23	23	Cyclohexanone	0.813	3.65
36	training	1.81	1.14	1.35	0.27	28	68	Cyclohexanone	1.141	7.10
37	training	1.69	2.92	1.35	0.49	35	113	Cyclohexanone	2.075	9.51

38	training	1.31	4.11	1.35	0.94	38	38	Cyclohexanone	2.692	6.80
39	training	1.19	2.33	1.35	0.72	43	5.5	Cyclohexanone	1.749	0.00
40	training	1.06	1.73	1.06	0.61	48	53	Cyclohexanone	1.372	5.77
41	training	1.94	3.05	1.28	0.16	13	83	THFA	2.220	0.00
42	training	1.56	3.05	1.19	0.38	18	98	THFA	2.191	0.00
43	training	1.44	0.55	1.28	0.83	23	23	THFA	0.891	0.00
44	training	1.81	1.14	1.28	0.27	28	68	THFA	1.211	0.00
45	training	1.69	2.92	1.28	0.49	35	113	THFA	2.145	0.00
46	training	1.31	3.05	1.28	0.94	38	38	THFA	2.203	0.00
47	training	1.19	2.33	1.28	0.72	43	5.5	THFA	1.819	0.00
48	training	1.06	1.73	1.06	0.61	48	53	THFA	1.456	0.00
49	training	1.94	0.85	1.94	0.16	13	83	EA	1.166	0.00
50	training	1.56	0.85	1.19	0.38	18	98	EA	1.010	0.00
51	training	1.44	0.55	1.31	0.83	23	23	EA	0.874	0.00
52	training	1.81	0.85	1.56	0.27	28	68	EA	1.089	0.00
53	training	1.69	0.85	1.44	0.49	35	113	EA	1.063	0.00
54	training	1.31	0.85	1.69	0.94	38	38	EA	1.101	0.00
55	training	1.19	0.85	1.81	0.72	43	5.5	EA	1.119	0.00
56	training	1.06	0.85	1.06	0.61	48	53	EA	0.970	0.00
57	training	2.00	1.00	1.50	0.20	28	20	DMSO	1.885	6.89
58	training	2.00	1.00	1.50	0.20	30	40	DMSO	1.885	4.91
59	training	2.00	1.00	1.50	0.20	30	60	DMSO	1.885	5.97
60	training	2.00	1.00	1.50	1.00	30	60	DMSO	1.890	6.05
61	Optimisation	1.25	1.00	1.00	0.37	33	14	DCM	1.006	29.21
62	Optimisation	1.21	0.62	1.00	0.95	45	34	DCE	0.922	49.01
63	Optimisation	1.00	0.77	1.00	0.90	29	56	DCM	0.879	27.31
64	Optimisation	1.86	1.23	1.58	0.81	20	4.65	DCM	1.261	55.83
65	Optimisation	1.00	0.85	1.00	0.48	26	36	DCM	0.917	33.38
66	Optimisation	1.30	0.68	1.07	0.87	3	28	DCM	0.858	31.03
67	Optimisation	1.56	5.12	1.52	0.53	26	18	DCE	3.403	38.28
68	Optimisation	1.67	1.99	1.47	0.63	32	48	DCE	1.747	54.22
69	Optimisation	1.65	3.44	1.48	0.79	27	32	DCE	2.514	61.89
70	Optimisation	1.12	3.74	1.17	0.36	30	38	DMPU	4.098	8.38
71	Optimisation	1.61	0.61	1.58	0.16	34	33	DCM	0.923	52.00
72	Optimisation	1.75	3.67	1.60	0.74	48	5	DCM	2.544	40.97
73	Optimisation	1.65	1.99	1.08	0.45	32	32	THF	1.690	0.79
74	Optimisation	1.86	1.53	1.55	0.88	27	58	DCE	1.528	45.42
75	Optimisation	1.89	3.28	1.14	0.52	47	6	THF	2.394	1.92
76	Optimisation	2.00	1.31	1.50	0.20	29	20	DCM	1.292	61.26
77	Optimisation	1.77	3.89	1.78	0.47	45	30	BnOH	3.482	3.48
78	Optimisation	2.00	2.00	1.00	0.20	29	40	MeCN	1.820	6.10
79	Optimisation	1.82	1.38	1.59	0.73	42	41	DCE	1.454	56.8

80	Optimisation	1.03	0.92	1.30	0.55	45	16	EtOH	1.182	0.87
81	Optimisation	2.00	1.29	1.50	0.20	28	20	DCM	1.277	63.98
82	Optimisation	1.20	2.98	1.06	0.52	45	46	DCE	2.173	35.02
83	Optimisation	2.00	1.15	1.50	0.20	31	12	DCE	1.320	55.62
84	Optimisation	1.40	3.67	1.58	0.18	31	25	DCM	2.524	39.03
85	Optimisation	2.00	1.15	1.50	0.20	29	60	DMF	1.395	11.93
86	Optimisation	1.83	2.98	1.38	0.95	30	4.35	DCE	2.260	53.24
87	Optimisation	1.88	2.75	1.56	0.44	41	13	DCE	2.173	70.31
88	Optimisation	1.78	3.82	1.01	0.45	34	36	MeCN	2.774	0.43
89	Optimisation	1.06	1.15	1.60	0.66	27	42	DCM	1.193	26.82
90	Optimisation	1.00	0.53	1.00	0.10	29	33	DCE	0.867	30.93

***Table S7**. Repeated experiments for reproducibility estimation.*

Rxn No	Alkene eq.	$Ir(ppy)_3$ mol%	HEH eq.	Acid eq.	T / C	Time / min	Solvent	Yield / %	Mean ± STD
1	2.00	1.00	1.50	0.20	30	20	DCM	50.38	
2	2.00	1.00	1.50	0.20	30	20	DCM	46.95	48.97 ± 1.79
3	2.00	1.00	1.50	0.20	30	20	DCM	49.58	

Table S8. *Predicted and measured Hantzsch ester solubility in various solvents.*

No	Solvent	COSMO*therm* predicted solubility / mM	Experimentally measured solubility / mM
1	formicacid	532.86	
2	hexamethylphosphoramide	174.29	
3	n-methyl-2-pyrrolidinone	172.00	390.82
4	n,n-dimethylacetamide	158.03	330.22
5	1,3-dimethyltetrahydropyrimidin-2(1h)-one	156.43	357.02
6	ch2cl2	118.66	64.11
7	dimethylformamide	99.22	178.33
8	1,3-dimethyl-2-imidazolidinone	91.88	309.25
9	thf	84.82	93.96
10	dioxane	82.09	51.66
11	propionicacid	76.79	25.64
12	chcl3	53.81	152.17
13	cyclopentanone	52.86	
14	aceticacid	51.05	41.75
15	1-methyl-pyrrolidine	50.29	
16	methanol	42.30	22.11
17	benzylalcohol	42.04	
18	cyclohexanone	41.51	
19	ethylacetate	40.79	80.56
20	methylacetate	40.25	
21	2-methyltetrahydrofuran	39.57	122.21
22	2-furanmethanol	36.82	86.45
23	tetrahydrofurfurylalcohol	36.74	
24	ethylformate	33.79	
25	propanone	31.65	49.71
26	dimethylsulfoxide	30.98	159.51
27	ethanol	30.28	45.02
28	propanol	29.95	
29	n-propylacetate	29.11	
30	isobutanol	29.07	
31	methylpropionate	28.92	30.08
32	1,2-dichloroethane	27.91	
33	1-methoxy2-propanol	27.76	
34	butanone	26.92	52.45
35	1-butanol	26.25	22.35
36	isopropylacetate	25.34	28.94
37	2-methoxyethanol	25.29	

38	isopentanol	25.27	
39	2-hydroxypropanoicacidethylester	24.62	
40	dimethoxymethane	24.25	52.80
41	diglyme	24.11	
42	methylformate	23.72	
43	1,2-dimethoxyethane	23.32	
44	1-pentanol	22.63	
45	dihydro-5-methyl-2(3h)-furanone	21.55	
46	dimethylcarbonate	21.13	
47	4-methyl-2-pentanone	20.38	
48	diethylcarbonate	19.63	17.76
49	benzene	19.43	
50	n-butylacetate	18.77	
51	aceticacid-2-methylpropylester	17.96	
52	tert-butylacetate	17.94	
53	2-butanol	17.44	
54	2-propanol	16.98	60.47
55	2-hexanone	16.70	
56	Dibutyl_Isosorbide_Ether	15.52	
57	1-heptanol	15.07	
58	2-methyl-2-butanol	14.86	
59	isoamylacetate	14.21	
60	lacticacid	14.08	
61	n-pentylacetate	13.69	
62	1-octanol	13.22	
63	ethylsuccinate	12.37	
64	dimethyl_adipate	11.85	
65	1,2-ethanedioldiacetate	11.61	
66	acetonitrile	11.25	19.00
67	methyl-t-butylether	10.79	
68	diethylether	10.25	3.95
69	cyrene	9.47	
70	anisole	9.01	
71	butylenecarbonate	8.49	
72	cyclopentyl-methyl-ether	7.98	
73	aceticanhydride	7.82	
74	chlorobenzene	7.16	
75	1,2-dichlorobenzene	6.90	10.10
76	toluene	6.80	4.74
77	glycerol-triacetate	6.63	
78	nitromethane	6.58	
79	Dibutyl_Succinate	6.40	
80	aceticacid-2-ethylhexylester	5.95	

81	propyleneglycol	5.94	
82	1-chlorobutane	5.93	
83	methyl-tert-amylether	5.85	
84	tetrahydro-2,2,5,5-tetramethylfuran	5.22	
85	propylenecarbonate	4.43	
86	1,3-propanediol	4.41	
87	diisopropylether	4.18	
88	1,2-dimethylbenzene	3.80	
89	1,4-dimethylbenzene	3.73	
90	2-ethoxy-2-methyl-propane	3.67	
91	1,3-dimethylbenzene	3.64	
92	trifluoromethylbenzene	2.96	
93	isopropylbenzene	2.73	
94	1-methyl-4-isopropylbenzene	1.79	
95	glycol	1.73	
96	cs2	1.58	
97	methyloleate	1.44	4.36
98	triethylamine	1.42	1.58
99	dipentene	1.20	
100	glycerol	0.55	0.00
101	cyclohexane	0.23	0.79
102	pentane	0.22	
103	methylcyclohexane	0.21	
104	hexane	0.18	0.04
105	2,2,4-trimethylpentane	0.18	
106	n-heptane	0.15	
107	h2o	0.04	0.79

References

1. Aillet, T.; Loubiere, K.; Dechy-Cabaret, O.; Prat, L., Accurate Measurement of the Photon Flux Received Inside Two Continuous Flow Microphotoreactors by Actinometry. *International Journal of Chemical Reactor Engineering* **2014,** *12* (1), 257-269.
2. Loponov, K. N.; Lopes, J.; Barlog, M.; Astrova, E. V.; Malkov, A. V.; Lapkin, A. A., Optimization of a Scalable Photochemical Reactor for Reactions with Singlet Oxygen. *Organic Process Research & Development* **2014,** *18* (11), 1443-1454.

3. Jespersen, D.; Keen, B.; Day, J. I.; Singh, A.; Briles, J.; Mullins, D.; Weaver, J. D., Solubility of Iridium and Ruthenium Organometallic Photoredox Catalysts. *Organic Process Research & Development* **2019,** *23* (5), 1087-1095.
